# The whole‐cell Ca^2+^ release‐activated Ca^2+^ current, *I*_CRAC_, is regulated by the mitochondrial Ca^2+^ uniporter channel and is independent of extracellular and cytosolic Na^+^

**DOI:** 10.1113/JP276551

**Published:** 2019-02-06

**Authors:** Krishna Samanta, Daniel Bakowski, Nader Amin, Anant B. Parekh

**Affiliations:** ^1^ Department of Physiology Anatomy and Genetics University of Oxford Sherrington Building, Parks Road Oxford OX1 3PT UK; ^2^ Chemistry Research Laboratory Department of Chemistry University of Oxford 12 Mansfield Road Oxford OX1 3TA UK

**Keywords:** calcium channel, Mitochondria, store‐operated

## Abstract

**Key points:**

- Ca^2+^ entry through Ca^2+^ release‐activated Ca^2+^ channels activates numerous cellular responses. Under physiological conditions of weak intracellular Ca^2+^ buffering, mitochondrial Ca^2+^ uptake regulates CRAC channel activity.
- Knockdown of the mitochondrial Ca^2+^ uniporter channel prevented the development of *I*_CRAC_ in weak buffer but not when strong buffer was used instead.
- Removal of either extracellular or intra‐pipette Na^+^ had no effect on the selectivity, kinetics, amplitude, rectification or reversal potential of whole‐cell CRAC current.
- Knockdown of the mitochondrial Na^+^–Ca^2+^ exchanger did not prevent the development of *I*_CRAC_ in strong or weak Ca^2+^ buffer.
- Whole cell CRAC current is Ca^2+^‐selective.
- Mitochondrial Ca^2+^ channels, and not Na^+^‐dependent transport, regulate CRAC channels under physiological conditions.

**Abstract:**

Ca^2+^ entry through store‐operated Ca^2+^ release‐activated Ca^2+^ (CRAC) channels plays a central role in activation of a range of cellular responses over broad spatial and temporal bandwidths. Mitochondria, through their ability to take up cytosolic Ca^2+^, are important regulators of CRAC channel activity under physiological conditions of weak intracellular Ca^2+^ buffering. The mitochondrial Ca^2+^ transporter(s) that regulates CRAC channels is unclear and could involve the 40 kDa mitochondrial Ca^2+^ uniporter (MCU) channel or the Na^+^–Ca^2+^–Li^+^ exchanger (NCLX). Here, we have investigated the involvement of these mitochondrial Ca^2+^ transporters in supporting the CRAC current (*I*_CRAC_) under a range of conditions in RBL mast cells. Knockdown of the MCU channel impaired the activation of *I*_CRAC_ under physiological conditions of weak intracellular Ca^2+^ buffering. In strong Ca^2+^ buffer, knockdown of the MCU channel did not inhibit *I*_CRAC_ development demonstrating that mitochondria regulate CRAC channels under physiological conditions by buffering of cytosolic Ca^2+^ via the MCU channel. Surprisingly, manipulations that altered extracellular Na^+^, cytosolic Na^+^ or both failed to inhibit the development of *I*_CRAC_ in either strong or weak intracellular Ca^2+^ buffer. Knockdown of NCLX also did not affect *I*_CRAC_. Prolonged removal of external Na^+^ also had no significant effect on store‐operated Ca^2+^ entry, on cytosolic Ca^2+^ oscillations generated by receptor stimulation or on CRAC channel‐driven gene expression. In the RBL mast cell, Ca^2+^ flux through the MCU but not NCLX is indispensable for activation of *I*_CRAC_.

## Key points

- Ca^2+^ entry through Ca^2+^ release‐activated Ca^2+^ channels activates numerous cellular responses. Under physiological conditions of weak intracellular Ca^2+^ buffering, mitochondrial Ca^2+^ uptake regulates CRAC channel activity.
- Knockdown of the mitochondrial Ca^2+^ uniporter channel prevented the development of *I*_CRAC_ in weak buffer but not when strong buffer was used instead.
- Removal of either extracellular or intra‐pipette Na^+^ had no effect on the selectivity, kinetics, amplitude, rectification or reversal potential of whole‐cell CRAC current.
- Knockdown of the mitochondrial Na^+^–Ca^2+^ exchanger did not prevent the development of *I*_CRAC_ in strong or weak Ca^2+^ buffer.
- Whole cell CRAC current is Ca^2+^‐selective.
- Mitochondrial Ca^2+^ channels, and not Na^+^‐dependent transport, regulate CRAC channels under physiological conditions.

## Introduction

CRAC channels are a major route for Ca^2+^ influx in eukaryotic cells where they regulate a variety of processes ranging from exocytosis to Ca^2+^‐dependent gene expression (Parekh, 2010). The CRAC channel is activated by depletion of the endoplasmic reticulum (ER) Ca^2+^ store (Hoth & Penner, 1992), the Ca^2+^ content of which is sensed by the ER‐resident STIM proteins (Prakriya & Lewis, 2015). Physiologically, stores are depleted following stimulation of cell‐surface receptors that couple to phospholipase C to increase the levels of the second messenger inositol 1,4,5‐trisphopshate (Ins*P*_3_) (Parekh & Putney, 2005). Ins*P*_3_ opens Ins*P*_3_‐gated Ca^2+^ channels in the ER membrane, leading to a loss of Ca^2+^ within the ER. Upon store depletion, STIM proteins oligomerize and the aggregates migrate across the ER to reach specialized regions of junctional ER that are juxtaposed with the plasma membrane (Hogan, 2015). At these ER–plasma membrane contact sites, STIM binds to and gates open Orai1 proteins, which are the pore‐forming subunits of the CRAC channel (Prakriya & Lewis, 2015). Classical biophysical studies from several groups have demonstrated that CRAC channels are very selective for Ca^2+^, with *P*_Ca_/*P*_Na_ > 1000, distinguish between different divalent cations and exhibit a minuscule unitary conductance, estimated from noise analysis to be in the low femtosiemens range (Parekh & Putney, 2005; Prakriya & Lewis, 2015).

Mechanisms that regulate CRAC channel activity impact on the time course of the Ca^2+^ signal and therefore on the extent of activation and duration of Ca^2+^‐dependent responses. Several mechanisms have been described including negative feedback by protein kinase C (Parekh & Penner, 1995) and by cytosolic Ca^2+^ itself. Ca^2+^ entry through CRAC channels activates two independent inhibitory pathways, resulting in fast and slow Ca^2+^‐dependent inactivation. Fast Ca^2+^‐dependent inactivation is a bi‐exponential process developing within milliseconds of Ca^2+^ entry and is driven by Ca^2+^ microdomains near each open channel (Zweifach & Lewis, 1995*a*; Fierro & Parekh, 1999*a*). It is a complex process with various domains of Orai1 and STIM1 implicated (Prakriya & Lewis, 2015). Slow Ca^2+^‐dependent inactivation develops over tens of seconds, requires a rise in bulk cytosolic Ca^2+^ and involves the ER‐resident protein SARAF (Zweifach & Lewis, 1995*b*; Parekh, 1998; Palty *et al*. 2012).

Mitochondria are also important regulators of CRAC channel activity through buffering of cytosolic Ca^2+^ (Rizzuto & Pozzan, 2006). Ca^2+^ entry into mitochondria is driven by the large negative potential across the inner mitochondrial membrane and occurs through the mitochondrial Ca^2+^ uniporter (MCU), a Ca^2+^‐selective inwardly rectifying channel spanning the inner mitochondrial membrane (Baughman *et al*. 2011; De Stefani *et al*. 2011). Other Ca^2+^ uptake pathways have also been reported, including one involving uncoupler proteins 2 and 3 (Trenker *et al*. 2007). In RBL mast cells, we have shown that mitochondria buffer Ins*P*_3_‐dependent Ca^2+^ release and this leads to more extensive store depletion (Gilabert & Parekh, 2000; Samanta *et al*. 2014). Mitochondria also buffer Ca^2+^ entry, reducing the rate and extent of Ca^2+^‐dependent slow inactivation of the channels (Gilabert & Parekh, 2000; Hoth *et al*. 2000; Glitsch *et al*. 2002b). However, the molecular identity of the mitochondrial Ca^2+^ channel necessary for supporting *I*_CRAC_ is unknown. Mitochondria can extrude Ca^2+^ from the matrix through the activities of two exchangers, the Ca^2+^–H^+^ exchanger LetM1 (Jiang *et al*. 2009) and the Na^+^–Ca^2+^–Li^+^ exchanger (NCLX) (Palty *et al*. 2010). Recently, it has been proposed that mitochondrial Ca^2+^ extrusion mediated by NCLX is essential for the activation of *I*_CRAC_ following store depletion (Ben‐Kassus Nissim *et al*. 2017). This mechanism was observed in high cytosolic BAPTA, suggesting it was independent of mitochondrial Ca^2+^ buffering. It was also proposed that store depletion activated plasma membrane Na^+^ channels in parallel with *I*_CRAC_ with the former driving the rise in cytosolic Na^+^ needed to stimulate mitochondrial NCLX and thus enabling *I*_CRAC_ to develop (Ben‐Kassus Nissim *et al*. 2017). Consequently, the whole‐cell CRAC current would involve a Na^+^ current, which questions earlier conclusions on the selectivity of the CRAC current. In this study, we address the role of the MCU channel and both extracellular and cytosolic Na^+^ in the regulation of *I*_CRAC_, Ca^2+^ signalling and CRAC channel‐driven gene expression. We find a central role for the MCU but not for extracellular or intracellular Na^+^ in supporting CRAC channel activity under physiological conditions.

## Methods

### Cell culture

Rat basophilic leukemia (RBL‐2H3) cells were purchased from ATCC (Manassas, VA, USA). Cells were cultured in Dulbecco's modified Eagle's medium (DMEM) supplemented with 10% fetal calf serum and 1% penicillin/streptomycin, as previously described (Samanta *et al*. 2014).

### Patch clamp recordings

Patch clamp experiments were conducted in the tight seal whole‐cell configuration at room temperature (20–24°C) as previously described (Glitsch *et al*. 2002b). Pipettes were pulled from borosilicate glass and were Sylgard‐coated and fire polished. Pipettes had resistances of 3–6 MΩ when filled with the various internal solutions used for *I*_CRAC_ measurements (Table 1). The CRAC current was measured by applying voltage ramps (−100 to +100 mV in 50 ms) at 0.5 Hz from a holding potential of 0 mV. Current through Kv1.5 channels was measured using voltage ramps spanning −100 to +100 mV in 250 ms, applied at 0.2 Hz to allow for recovery from C‐type inactivation between ramps. Holding potential for recording of Kv1.5 was −80 mV. Currents were filtered using an eight‐pole Bessel filter at 2.5 kHz and digitized at 100 μs. Capacitative currents were compensated before each ramp or step by using the automatic compensation of the EPC 9‐2 amplifier (HEKA, Lambrecht/Pfalz, Germany). Leak currents were subtracted by averaging three to five ramp currents obtained just before *I*_CRAC_ had started to develop (passive store depletion) or by subtracting the first one or two ramp currents (active store depletion with Ins*P*_3_). Recordings were made under paired conditions, in that test conditions and controls were carried out on alternate coverslips on the same days.

**Table 1:** Solutions used

Solution	Composition (mM)
Internal solutions	
Na^+^ containing, active store depletion	145 Cs‐glutamate, 8 NaCl, 1 MgCl_2_, 10 HEPES, 10 EGTA, 0.03 Ins*P*_3_, pH 7.2 (CsOH)
Na^+^ free, active store depletion	145 Cs‐glutamate, 8 CsCl, 1 MgCl_2_, 10 HEPES, 10 EGTA, 0.03 Ins*P*_3_, pH 7.2 (CsOH)
Na^+^ containing, passive store depletion	145 Cs‐glutamate, 8 NaCl, 1 MgCl_2_, 10 HEPES, 10 EGTA, pH 7.2 (CsOH)
Na^+^ free, passive store depletion	145 Cs‐glutamate, 8 CsCl, 1 MgCl_2_, 10 HEPES, 10 EGTA, pH 7.2 (CsOH)
Na^+^ containing, mitochondrial cocktail	145 Cs‐glutamate, 8 NaCl, 1 MgCl_2_, 10 HEPES, 0.35 EGTA, 0.03 Ins*P*_3_, 2 pyruvic acid, 2 K‐malate, 1 KH_2_PO_4_, 2 Mg‐ATP, pH 7.2 (CsOH)
Na^+^‐free, mitochondrial cocktail	145 Cs‐glutamate, 8 CsCl, 1 MgCl_2_, 10 HEPES, 0.35 EGTA, 0.03 Ins*P*_3_, 2 pyruvic acid, 2 K‐malate, 1 KH_2_PO_4_, 2 Mg‐ATP, pH 7.2 (CsOH)
Kv1.5, K^+^ based	145 K‐glutamate, 8 NaCl, 1 MgCl_2_, 10 HEPES, 0.35 EGTA, 2 Mg‐ATP, pH 7.2 (KOH)
Kv1.5, Cs^+^ based	145 Cs‐glutamate, 8 NaCl, 1 MgCl_2_, 10 HEPES, 0.35 EGTA, 2 Mg‐ATP, pH 7.2 (CsOH)
Extracellular solutions for patch clamp recordings	
Na^+^ containing	155 NaCl, 10 CaCl_2_, 10 CsCl, 2.8 KCl, 2 MgCl_2_, 10 HEPES, 10 glucose, pH 7.4 (NaOH)
Na^+^ free	155 Tris‐Cl, 10 CaCl_2_, 10 CsCl, 2.8 KCl, 2 MgCl_2_, 10 HEPES, 10 glucose, pH 7.4 (CsOH)
Ca^2+^ free	Ca^2+^‐free solution was made by omitting CaCl_2_ from the above solutions
Kv1.5	155 NaCl, 2 CaCl_2_, 10 CsCl, 2.8 KCl, 2 MgCl_2_, 10 HEPES, 10 glucose, pH 7.4 (NaOH)
Extracellular solutions for fluorescence measurements	
Na^+^ containing	155 NaCl, 2 CaCl_2_, 2.8 KCl, 2 MgCl_2_, 10 HEPES, 10 glucose, pH 7.4 (NaOH)
Na^+^ free	155 Tris‐Cl, 2 CaCl_2_, 2.8 KCl, 2 MgCl_2_, 10 HEPES, 10 glucose, pH 7.4 (HCl).
Ca^2+^ free	Ca^2+^‐free solution was made by omitting CaCl_2_ from the above solutions followed by addition of 0.1 mM EGTA. pH was returned to 7.4 with either NaOH or HCl.

The solutions used are listed in Table 1.

### ^23^Na nuclear magnetic resonance measurements

All sodium nuclear magnetic resonance (NMR) data were acquired at 132 MHz at room temperature using a Bruker Avance III NMR spectrometer (Billerica, MA, USA) equipped with a TBO probe. All experiments used a standard 90‐degree pulse‐acquire protocol with constant parameters, including number of scans, receiver gain and pulse length. All samples for NMR were prepared to a final volume of 0.6 mL in 5 mm NMR tubes. Calibration curve samples were prepared in 100% D_2_O, and samples for analysis were prepared with 10% D_2_O.

A calibration curve for free sodium concentration was generated using five different NaCl samples with sodium concentrations in the range 0.02–340 mM. Peak integrals were plotted against concentration, and the calibration curve showed excellent linearity (*R* ^2^ = 0.9998). To determine the free sodium concentration in a sample, the sodium NMR peak was integrated and fitted to the curve.

### Cytosolic Ca^2+^ measurements

Cytosolic Ca^2+^ measurements were carried out at room temperature using the IMAGO charge‐coupled device camera‐based system from TILL Photonics (now FEI GmbH, Munich, Germany), as described previously (Samanta *et al*. 2014). Cells were loaded with Fura‐2/AM (1 μM; Molecular Probes/Thermo Fisher Scientific, Waltham, MA, USA) for 40 min in the dark at room temperature and then washed three times in standard external solution (Table 1). Cells were left for 15 min to allow further de‐esterification. Cells were alternately excited at 356 nm and 380 nm (20 ms exposures) at 0.5 Hz. Analysis was performed offline using Igor Pro for Windows (WaveMetrics, Lake Oswego, OR, USA). Ca^2+^ signals are plotted as *R*, denoting the 356/380 ratio.

### Cytosolic Na^+^ measurements

Cells were loaded with CoroNa Green‐AM (5 μM; Invitrogen, Carlsbad, CA, USA) for 45 min at room temperature in the dark in either standard Na^+^‐containing external solution or Na^+^‐free external solution, as described in the text and then washed three times in the appropriate Na^+^‐containing or Na^+^‐free solution. Cells were left for 15 min to allow further de‐esterification. Cells were excited at 490 nm (20 ms exposures) and images were acquired every 3 s. Images were analysed offline using Igor Pro. The Na^+^ signals were normalized to the averaged baseline signal (*F*/*F*_0_), obtained at the beginning of the measurements. In some experiments, gramicidin A (Santa Cruz Biotechnology, Dallas, TX, USA; cat. no.: SC‐203061) was added to raise cytosolic Na^+^. In some cells, CoroNa green signals bleached rapidly. Therefore, we measured the background rate of bleaching prior to stimulation and used only those cells which showed a gradual decay in fluorescence prior to stimulation. Two to three cells were used per field of view and therefore *n* = 9 (see text) indicates data from three to four separate experiments.

### Cell transfection and small interfering RNA

RBL cells were transfected using the AMAXA system, using nucleofector cell line kit V solution (from Lonza, Slough, UK, cat. no. VCA‐1003) and program T‐30 as described (Samanta *et al*. 2014).

Rat MCU SiRNA was from Origene (Rockville, MD, USA, cat. no.: SR508660) and had the following sequence:

SR508660A – rCrCrUrArGrArGrArArArUrArCrArAr UrCrArArCrUrCrAAG
SR508660B – rGrGrCrArGrArArArUrGrGrArUrCrUr UrArArGrArGrArCTG
SR508660C – rGrCrCrArGrArGrArCrArGrArCrArAr UrArCrUrUrArUrUAT

Rat NCLX small interfering RNA (siRNA) was from Invitrogen (cat. no. 10620310) and had the following sequence: AACGGCCACUCAACUGUCU.

Kv1.5 was a kind gift from Prof. Stefan Heinemann (Friedrich‐Schiller‐Universität Jena, Jena).

### c‐fos RT‐PCR

RBL cells were initially placed in either Na^+^‐containing or Na^+^‐free extracellular solution for 1 h (see text). Cells that had been placed in Na^+^‐free solution were then stimulated with a submaximal dose of leukotriene C_4_ (LTC_4_) (160 nM) or 2 μM thapsigargin for 10 min in Na^+^‐free solution and then washed with Na^+^‐free and Ca^2+^‐free solution (containing 0.1 mM EGTA) without stimulus for a further 40 min. For cells placed in Na^+^‐containing external solution, stimulation occurred in Na^+^‐containing solution for 10 min and then cells were washed with Na^+^‐containing and Ca^2+^‐free solution (with 0.1 mM EGTA) for 40 min without stimulus. Thereafter, for both groups (exposed to Na^+^‐containing or Na^+^‐free solution), total RNA was extracted by using an RNeasy Mini Kit (Qiagen, Hilden, Germany) (Ng *et al*. 2009). RNA was quantified spectrophotometrically by absorbance at 260 nm. Total RNA (1 μg) was reverse‐transcribed using the iScriptTM cDNA Synthesis Kit (Bio‐Rad Laboratories, Hercules, CA, USA), according to the manufacturer's instructions. Following cDNA synthesis, PCR amplification was performed using BIOX‐ACTTM ShortDNAPolymerase (Biolone) with primers specific for the detection of c‐fos and β‐actin from Invitrogen:

c‐Fos; forward: 5′ AGCCGACTCCTTCTCCAGCAT 3′
c‐Fos; reverse: 5′ CAGATAGCTGCTCTACTTTGC 3′
β‐actin; forward: 5′ TTGTAACCAACTGGGACGATATG 3′
β‐actin; reverse: 5′ GATCTTGATCTTCATGGTGCTAGG 3′

The PCR products were electrophoresed through an agarose gel and visualized by ethidium bromide staining.

### Gene expression assay

Twenty‐four hours after transfection with enhanced green fluorescent protein (EGFP)‐based reporter plasmid containing an NFAT promoter (gift from Dr Yuri Usachev, Iowa University), cells were placed in either Na^+^‐containing or Na^+^‐free extracellular solution for 1 h in an incubator and then stimulated with LTC_4_ or thapsigargin (100 nM) for 10 min in appropriate Na^+^‐containing or Na^+^‐free extracellular solution. Control groups had the same treatment but without stimulus. Cells were then washed to remove agonist and then maintained in either Na^+^‐containing or Na^+^‐free extracellular solution for a further 30 min (a time within which cells had recovered fully from stimulation with LTC_4_, as evidenced by a return to a stable resting cytosolic Ca^2+^ level) before washing with DMEM. Cells were left in the incubator for ∼24 h prior to detection of EGFP, as described (Kar *et al*. 2012b).

### Statistics

Statistical significance was calculated using the Instat 2.03 programme (for Macintosh; GraphPad Software, La Jolla, CA, USA). Comparison of two means was evaluated by Student's unpaired, two‐tailed *t* test. For comparison of more than two means, a two‐tailed ANOVA was used and the Student–Newman–Keuls or Bonferroni test was used for *post hoc* analyses. Data are presented as means ± SEM. The number of cells analysed is given in the figure legend and represents combined data from at least two independent cell preparations. Level of significance is shown as *P < 0.01 and ** *P* < 0.001.

## Results

### *I*_CRAC_ activated following store depletion with Ins*P*_3_ is unaffected by removal of external Na^+^

**Figure 1:**
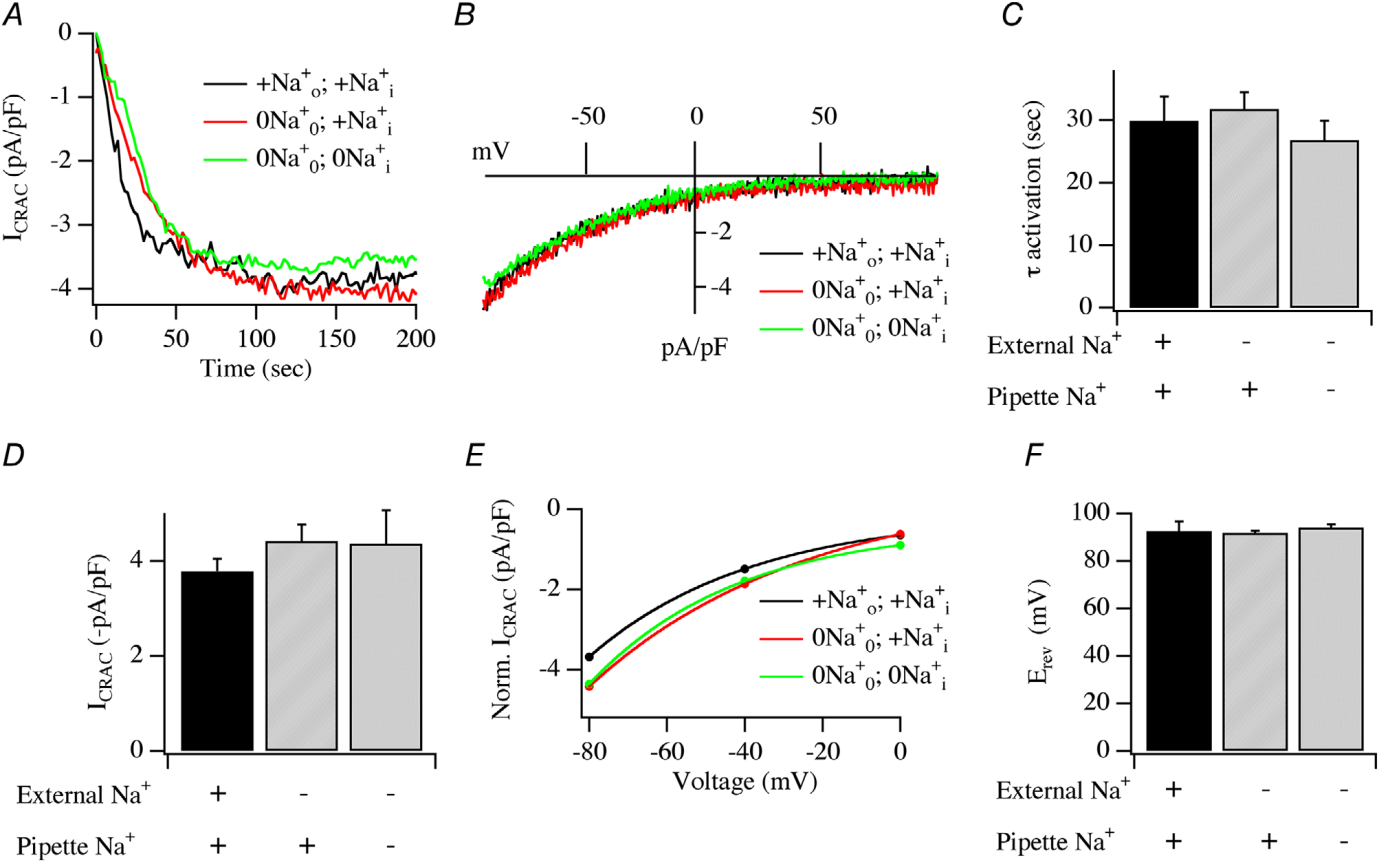
Impact of changing extracellular and intra‐pipette Na^+^ on *I*_CRAC_ evoked by Ins*P*_3_ *A*, time course of *I*_CRAC_ compared in different cells exposed to the different Na^+^ solutions indicated. $+\;{\mathrm{Na}}_{0}^{+};\;+\;{\mathrm{Na}}_{i}^{+}$ (black trace) denotes Na^+^‐containing external and pipette solutions. $0\;{\mathrm{Na}}_{0}^{+};\;+\;{\mathrm{Na}}_{i}^{+}$ (red trace) denotes Na^+^‐free external solution and Na^+^‐containing pipette solution. $0\;{\mathrm{Na}}_{0}^{+};\;0\;{\mathrm{Na}}_{i}^{+}$ (green trace) denotes Na^+^‐free external and pipette solutions. The cell in $0\;{\mathrm{Na}}_{0}^{+};\;+\;{\mathrm{Na}}_{i}^{+}$ was in Na^+^‐free solution for 27 min prior to break‐in. The cell in $0\;{\mathrm{Na}}_{0}^{+};\;0\;{\mathrm{Na}}_{i}^{+}$ was in Na^+^‐free solution for 19 min before break‐in. *B*, *I–V* relationships compared for the conditions indicated, taken once the whole‐cell currents in panel *A* had peaked. *C*, mono‐exponential time constant of activation of *I*_CRAC_ compared for the conditions shown. *D*, peak amplitude of *I*_CRAC_ compared for conditions indicated. *E*, the extent of inward rectification over the voltage range −80 mV to 0 mV depicted for the various conditions. *F*, bar chart comparing the reversal potential of *I*_CRAC_ for the different conditions. Averaged data for $+\;{\mathrm{Na}}_{0}^{+};\;+\;{\mathrm{Na}}_{i}^{+}$ was from 12 cells, $0\;{\mathrm{Na}}_{0}^{+};\;+\;{\mathrm{Na}}_{i}^{+}$ was from 9 cells and $0\;{\mathrm{Na}}_{0}^{+};\;0\;{\mathrm{Na}}_{i}^{+}$ was from 10 cells. There were no statistically significant differences between any of the groups.

To see whether extracellular Na^+^ was required for, or contributed to, *I*_CRAC_, we placed cells in Na^+^‐free external solution for at least 15 min prior to the onset of whole‐cell patch clamp recording. Stores were depleted by inclusion of Ins*P*_3_ together with 10 mM EGTA in a Na^+^‐containing patch pipette solution. In control cells maintained in Na^+^‐containing external solution, store depletion resulted in the development of *I*_CRAC_ (Fig. 1 *A*, black trace). The current–voltage (*I–V*) relationship exhibited the hallmarks of *I*_CRAC_ including non‐voltage‐dependent gating, marked inward rectification and a very positive reversal potential (Fig. 1 *B*). The current activated mono‐exponentially with a time constant of ∼25 s (Fig. 1 *C*) to reach an amplitude of ∼−3.5 pA/pF (Fig. 1 *D*). We displayed the extent of rectification by plotting the size of the current at −80, −40 and 0 mV (Fig. 1 *E*). The reversal potential of the current was ∼+90 mV (Fig. 1 *F*). In the absence of external Na^+^, *I*_CRAC_ developed in a manner that was qualitatively very similar to that seen in the presence of external Na^+^ (Fig. 1 *A*, red trace). The *I–V* relationship (Fig. 1 *B*), the mono‐exponential activation time constant (Fig. 1 *C*), the amplitude of the current (Fig. 1 *D*), the extent of rectification (Fig. 1 *E*) and the reversal potential (Fig. 1 *F*) were all indistinguishable from responses obtained in the presence of external Na^+^.

### Removal of intracellular Na^+^ does not affect *I*_CRAC_

Although the preceding experiments show that removal of external Na^+^ has no discernible impact on *I*_CRAC_, we hypothesized that the Na^+^ concentration in our pipette solution was sufficient to maintain cytosolic Na^+^ at the level needed to support the current. To test this, we repeated these experiments but now in the complete absence of Na^+^. We simultaneously used Na^+^‐free pipette and extracellular solutions. Under these conditions, *I*_CRAC_ developed with virtually identical features of time course, *I–V* relationship, amplitude, extent of rectification and reversal potential compared with cells dialysed with Na^+^‐containing external and pipette solutions (Fig. 1 *A*–*F*). None of the parameters measured were significantly affected by the absence of either extracellular Na^+^ or both extracellular and intracellular Na^+^. *I*_CRAC_ therefore activates robustly even when Na^+^ is absent simultaneously from extracellular and pipette solutions.

### Na^+^ concentrations in the solutions

**Figure 2:**
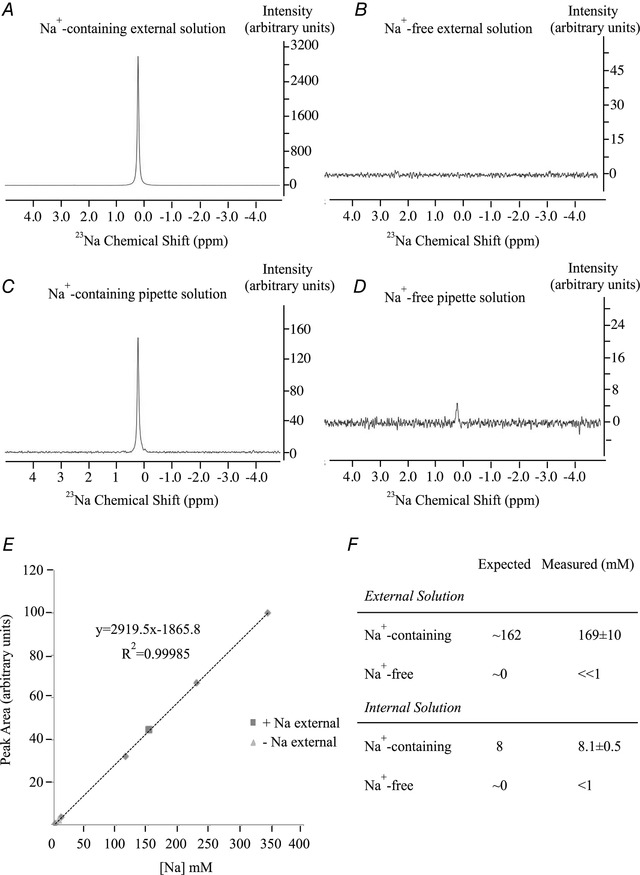
Measurement of Na^+^ in solutions using ^23^Na‐NMR *A*, spectrum for ^23^Na in standard external solution. *B*, ^23^Na spectrum in Na^+^‐free external solution. *C*, ^23^Na spectrum in standard Na^+^‐containing pipette solution. *D*, ^23^Na spectrum in Na^+^‐free pipette solution. *E*, calibration curve measuring peak area for different Na^+^ concentrations. Data from panels *A* and *C* are included. *F*, summary of Na^+^ concentrations in our solutions.

To confirm our Na^+^‐free solutions lacked Na^+^, we measured the Na^+^ concentrations using ^23^Na NMR. A strong sodium signal was detected in Na^+^‐containing external solution (Fig. 2 *A*) but no signal was seen in Na^+^‐free external solution (Fig. 2 *B*). A Na^+^ signal was also seen in our Na^+^‐containing pipette solution (Fig. 2 *C*) and this was almost abolished in the Na^+^‐free pipette solution (Fig. 2 *D*). We constructed a calibration curve (Fig. 2 *E*), and found the peak area of the ^23^Na NMR signal was linearly proportional to the ambient Na^+^ concentration over the range 1–350 mM. Our Na^+^‐containing external solution had a Na^+^ concentration of ∼169 mM (Fig. 2 *F*). Importantly, no Na^+^ was detected in our Na^+^‐free external solution indicating a Na^+^ concentration <<1 mM (Fig. 2 *F*). Our internal solution contained 8.1 mM Na^+^ (expected Na^+^ was 8 mM) and our Na^+^‐free pipette solution contained < 1 mM (Fig. 2 *F*). Therefore our Na^+^‐free solutions indeed were devoid of Na^+^.

### Time course of washout of a monovalent cation

**Figure 3:**
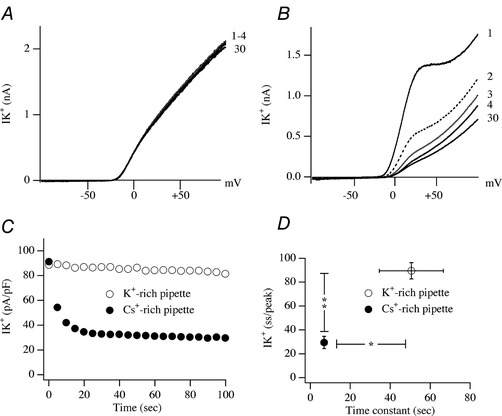
Rundown of Kv1.5 current is used as an indicator of the kinetics of monovalent cation exchange between pipette and cytosol *A*, *I–V* curves for a cell dialysed with a K^+^‐rich pipette solution are shown following application of the first 4 voltage ramps after break‐in and then the 30th ramp. Voltage ramps were applied every 5 s and the first ramp was given immediately upon break‐in. *B*, as in panel *A*, but a Cs^+^‐rich pipette solution was used instead. *C*, plot of the time course of the current from the two cells shown in panels *A* and *B*. Current was measured at +80 mV. *D*, plot of the steady state current/peak amplitude against the time constant for reaching steady state (6 cells for each condition). Steady state current was reached typically after ∼60 s and peak current was the amplitude of the current obtained from the first voltage ramp. Error bars for the time constant obtained with a Cs^+^‐rich pipette are contained within the symbol.

Freely diffusible ions or molecules that are present in the cytosol but omitted from the pipette solution will dialyse or wash out from the cytosol at a rate determined by the series resistance, cell size and molecular mass of the substance (Pusch and Neher, 1988). When Na^+^ is absent from the pipette solution, as was the case in the preceding experiments, the time constant for washout is expected to be fast, being ∼30 s with our typical series resistance of 8 MΩ and cell size of 10 pF. RBL cells, like many non‐excitable cells, do not express voltage‐gated Na^+^ channels and therefore it is not possible to measure the time course of Na^+^ dialysis through a shift in reversal potential of such channels. RBL cells do express a GTP‐activated Na^+^ channel but the current develops slowly over several minutes (Parekh, 1996), precluding a measure of the rate of Na^+^ washout. To estimate the kinetics of dialysis of a monovalent cation between cytosol and pipette solution, we therefore measured the rate of loss of K^+^ ions as gauged through the rate of rundown of voltage‐gated Kv1.5 currents following whole‐cell dialysis with a K^+^‐free pipette solution (potassium glutamate had been replaced by caesium glutamate) compared with a K^+^‐rich solution. Expression of Kv1.5 channels resulted in large outward K^+^ currents, measured using a voltage ramp protocol (Fig. 3 *A*). These currents were relatively stable because repetitive voltage ramps evoked similarly sized currents. Currents to the first four ramps after break‐in with a K^+^‐rich pipette solution are shown in Fig. 3 *A* along with that to the 30th ramp. The time course for this whole‐cell current (measured at +80 mV) is shown in Fig. 3 *C*. The K^+^ current was stable, declining <10% after 100 s of whole‐cell dialysis. Aggregate data comparing the extent of decline of the current with the time constant taken to reach the steady state level is shown in Fig. 3 *D*. The currents ran down considerably more quickly when K^+^ was replaced by Cs^+^ (Fig. 3 *B*–*D*). Rundown was rapid but reached a steady state where small Cs^+^ currents could be measured, reflecting the low *P*_Cs_/*P*_K_ of the channels (Fig. 3 *B* and *D*; Lin *et al*. 2001). Interaction between K^+^ and Cs^+^ shortly after break‐in likely accounts for the biphasic current that was seen only in the initial ramp currents (Fig. 3 *B*). Therefore monovalent group 1 cations like K^+^ and Cs^+^ exchange quickly between cytosol and pipette and, when omitted from the pipette solution, the smaller Na^+^ should wash out rapidly within a few tens of seconds.

### Robust *I*_CRAC_ activation by passive store depletion in the absence of Na^+^

The expected τ_washout_ of cytosolic Na^+^ is ∼30 s, when RBL cells are dialysed with our Na^+^‐free (<<1 mM) pipette solution. Because *I*_CRAC_ activates following dialysis with Ins*P*_3_ with a delay <2 s and with a time constant of activation of ∼25 s (Fig. 1 *C*), the current develops following dialysis with a Na^+^‐free pipette solution as intracellular free Na^+^ is declining quickly but is nevertheless still present. In order to activate *I*_CRAC_ after cytosolic Na^+^ had washed out of the cytosol, we depleted stores passively by dialysing cells with a pipette solution containing 10 mM EGTA but without Ins*P*_3_. Passive store depletion with EGTA activates *I*_CRAC_ slowly (Fierro & Parekh, 1999*b*); following a delay of ∼80 s, the current develops gradually, taking a further ∼200 s before reaching its peak value. Therefore with passive store depletion, cytosolic Na^+^ would have washed out of the cell following dialysis with a Na^+^‐free pipette solution well before *I*_CRAC_ activates.

**Figure 4:**
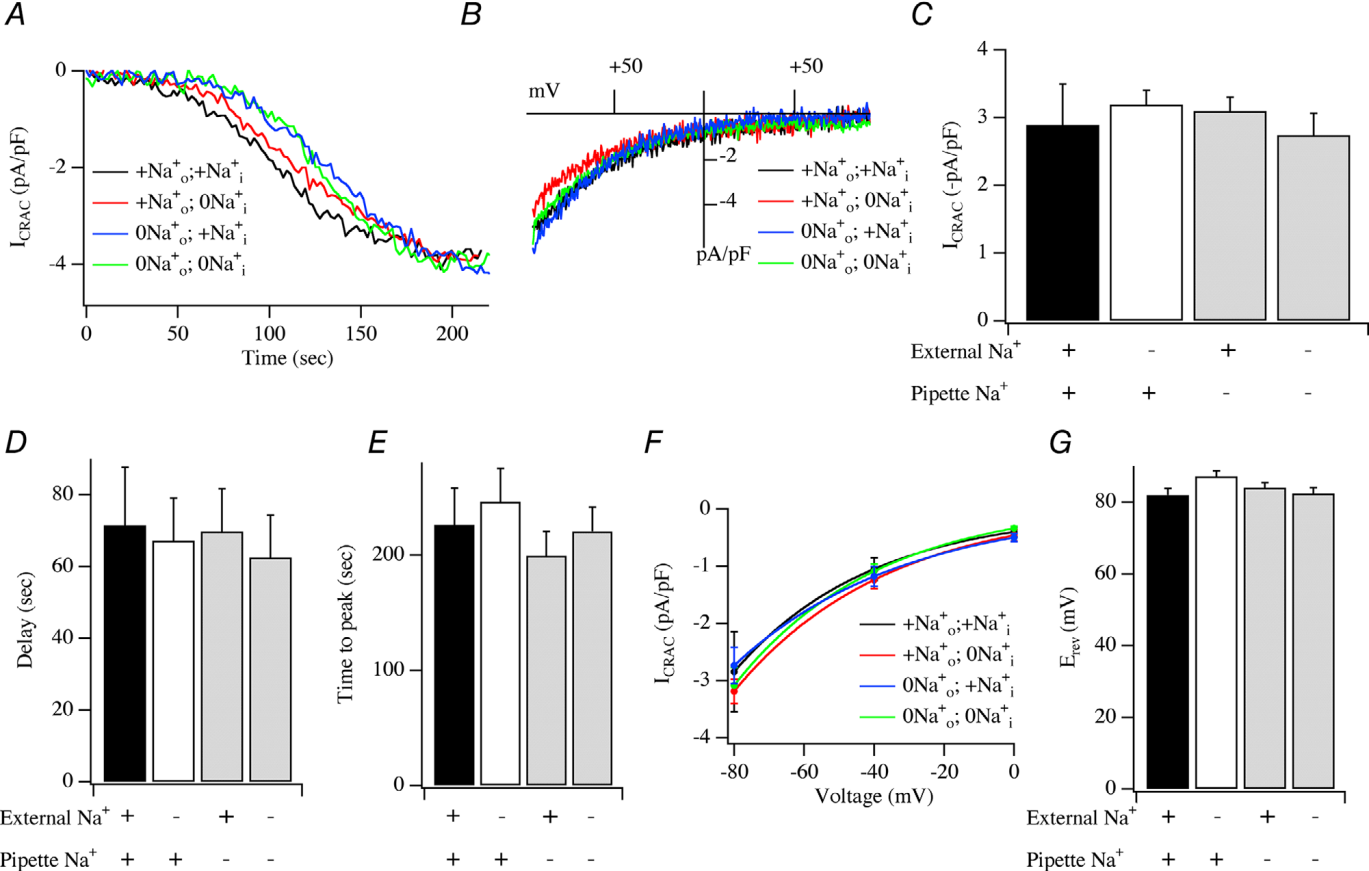
Effects of varying extracellular and intra‐pipette Na^+^ on *I*_CRAC_ evoked by passive store depletion by dialysis with high EGTA‐containing pipette solution *A*, time course of *I*_CRAC_ compared in the presence of different external and intra‐pipette Na^+^ concentrations. *B*, steady‐state *I–V* curves taken from experiments in panel *A*. *C*, peak amplitude of *I*_CRAC_ compared for the conditions shown. *D*, delay before *I*_CRAC_ activates compared for the conditions indicated. *E*, time to peak (measured from the delay) shown for the various experimental conditions. *F*, inward rectification, over the voltage range −80 to 0 mV is compared. *G*, bar chart showing reversal potential of the whole‐cell current for the different conditions. Averaged data for $+\;{\mathrm{Na}}_{0}^{+};\;+\;{\mathrm{Na}}_{i}^{+}$ was from 12 cells, $0\;{\mathrm{Na}}_{0}^{+};\;+\;{\mathrm{Na}}_{i}^{+}$ was from 10 cells, $+\;{\mathrm{Na}}_{0}^{+};\;0\;{\mathrm{Na}}_{i}^{+}$ was from 8 cells and $0\;{\mathrm{Na}}_{0}^{+};\;0\;{\mathrm{Na}}_{i}^{+}$ was from 11 cells. There were no statistically significant differences between the groups.

In control cells exposed to Na^+^‐containing extracellular solution and dialysed with a pipette solution containing Na^+^, *I*_CRAC_ activated slowly as expected for passive store depletion (Fig. 4 *A*, black trace). The *I–V* relationship exhibited the characteristics of *I*_CRAC_ (Fig. 4 *B*). The current reached an amplitude of ∼−3 pA/pF (Fig. 4 *C*), similar to that evoked by Ins*P*_3_ (Fig. 1 *D*). *I*_CRAC_ developed gradually, exhibiting a delay of ∼75 s (Fig. 4 *D*) and the subsequent time taken for the current to peak was ∼200 s (Fig. 4 *E*). The extent of rectification (Fig. 4 *F*) and reversal potential (Fig. 4 *G*) were similar to those seen when Ins*P*_3_ was used to deplete the stores instead (Fig. 1 *E* and *F*).

We systematically removed extracellular Na^+^ and pipette Na^+^ to see whether these manoeuvres affected any of the properties of *I*_CRAC_. Removal of pipette Na^+^, leaving extracellular Na^+^ present, or removal of extracellular Na^+^ leaving pipette Na^+^ intact, had no effect on any of the properties of the current activated by passive store depletion (Fig. 4 *A*–*G*). We also activated *I*_CRAC_ through passive store depletion in the simultaneous absence of intra‐ and extracellular Na^+^. In cells pre‐exposed to Na^+^‐free external solution for 15–30 min and then dialysed with a Na^+^‐free pipette solution, *I*_CRAC_ was indistinguishable from that seen in the presence of extracellular and intra‐pipette Na^+^ (Fig. 4 *A*–*G*). None of the kinetics of activation, the *I–V* relationship, the amplitude, the extent of rectification of the current or the reversal potential were affected by the simultaneous removal of Na^+^ from both extracellular and pipette solutions.

### Ca^2+^‐dependent fast inactivation of CRAC channels

**Figure 5:**
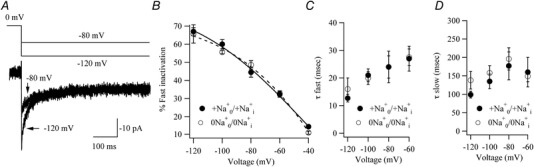
Effect of removing Na^+^ on Ca^2+^‐dependent fast inactivation of CRAC channels *A*, fast inactivation compared following hyperpolarizing pulses to −120 and −80 mV. *B*, aggregate data comparing the percentage of fast inactivation with hyperpolarizing pulse potential compared for the two conditions shown. Each point is the mean of 5 cells. Extent of fast inactivation was assessed as (1 − steady‐state current at end of pulse/peak current) ×100%. *C*, graph comparing the fast time constant of fast inactivation between Na^+^‐containing and Na^+^‐free solutions. *D*, graph comparing the slow time constant of fast inactivation.

Another hallmark of CRAC channels is that they exhibit Ca^2+^‐dependent fast inactivation whereby Ca^2+^ ions that have permeated a channel feed back to reduce further channel activity. Fast inactivation develops along a biexponential time course during hyperpolarizing pulses below −40 mV. In RBL cells, we have previously characterized fast inactivation in detail (Fierro & Parekh, 1999*a*) and found it exhibits identical features to those first described in T cells (Zweifach and Lewis, 1995*a*). If a Na^+^ current developed in parallel with *I*_CRAC_, a simple prediction would be that the rate and/or extent of fast inactivation of *I*_CRAC_ should be altered by removal of external Na^+^ as it is unlikely the Na^+^‐permeable channels would show identical rates and extents of fast inactivation to those of CRAC channels. To test this, we compared fast inactivation between cells exposed to Na^+^‐containing extracellular solution with those maintained in Na^+^‐free solution for several minutes prior to the onset of experiments. Na^+^‐free pipette solution was used in both cases. Fast inactivation was induced by application of voltage steps (−40 mV to −120 mV in 20 mV increments, 250 ms duration). In control cells maintained in Na^+^‐containing solution, hyperpolarizing pulses below −40 mV elicited fast inactivation which increased with stronger hyperpolarization (Fig. 5 *A*). The extent of fast inactivation increased monotonically with hyperpolarizing pulse potential (Fig. 5 *B*) and could be fitted with a Boltzmann‐type equation (where *R*, *T*, *Z* and *F* have their usual meanings)

$$\mathrm{CDI}=\frac{{\mathrm{CDI}}_{\max}}{1-\exp\frac{zdF}{RT}\left(V_{1/2}\right)}$$

yielding *V*_1/2_ of −68 mV (Fig. 5 *B*). Inactivation followed a biexponetial process, with time constants of ∼10 and ∼100 ms at −120 mV for the fast and slow components, respectively (Fig. 5 *C* and *D*).

In Na^+^‐free external solution, fast inactivation showed a superimposable dependence on hyperpolarizing pulse potential (Fig. 5 *B*), with a *V*_1/2_ of −66 mV. The biexponential kinetics of inactivation in Na^+^‐free solution were also similar to those seen in Na^+^‐containing solution (Fig. 5 *C* and *D*).

### *I*_CRAC_ activates after extensive dialysis of the cytosol with Na^+^‐free solutions

**Figure 6:**
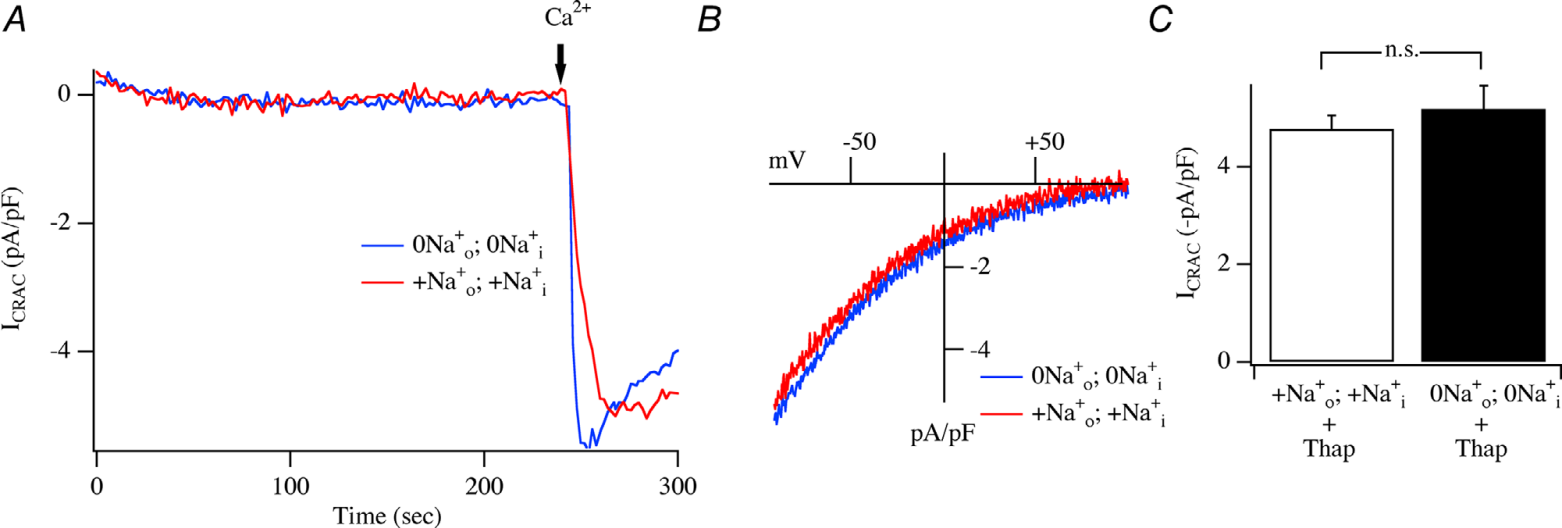
Activation of *I*_CRAC_ following extensive dialysis of the cytosol with Na^+^‐free pipette solution *A*, traces comparing time course of *I*_CRAC_ following dialysis with either Na^+^‐free external and pipette solutions (labelled $0\;{\mathrm{Na}}_{0}^{+};\;0\;{\mathrm{Na}}_{i}^{+}$) or Na^+^‐containing solutions ($+\;{\mathrm{Na}}_{0}^{+};\;+\;{\mathrm{Na}}_{i}^{+}$). Cells were kept in Na^+^‐ and Ca^2+^‐free extracellular solution for ∼10 min prior to break‐in. Blue trace shows *I*_CRAC_ after dialysis with Na^+^‐free pipette solution for ∼220 s before external Ca^2+^ was readmitted. Red trace is a control recording taken in the presence of Na^+^‐containing extracellular and pipette solutions. *B*, *I–V* curves, taken once the currents in panel *A* had peaked. *C*, amplitude of *I*_CRAC_ compared for the conditions shown. Each bar is the mean of between 6 and 9 cells. No significant difference was found between the 2 groups (*P* > 0.1). No significant difference in peak amplitude was seen with these various times of Ca^2+^ readmission (applied ∼60‐220 s after break‐in) and all recordings for each condition have been combined.

We designed experiments to activate *I*_CRAC_ after extensive dialysis of the cytosol with Na^+^‐free pipette solution under physiological conditions of weak intracellular Ca^2+^ buffering. First, cells were placed in Na^+^‐ and Ca^2+^‐free external solution supplemented with the sarco/endoplasmic reticulum Ca^2+^‐ATPase (SERCA) pump blocker thapsigargin (2 μM). By blocking SERCA pumps, thapsigargin prevents store refilling and, in the continuous presence of Ca^2+^ leak out of the ER, the stores deplete and CRAC channels open. But in the absence of external Ca^2+^, no current flows. Cells were dialysed with a Na^+^‐free pipette solution containing weak Ca^2+^ buffer supplemented with a mitochondrial cocktail solution that maintains the organelle in an energized state and therefore sustains mitochondrial Ca^2+^ buffering (Gilabert & Parekh, 2000). We selected cells with a low series resistance (typically <8MΩ) and small size (input capacitance typically <10 pF) in order to achieve rapid dialysis of Na^+^ from the cytosol. Following break‐in, no current developed. We dialysed a cell with a Na^+^‐free pipette solution for ∼220 s (∼8 times > than τ_washout_ of cytosolic Na^+^) before readmitting external Ca^2+^; a robust CRAC current was still produced (Fig. 6 *A*; blue trace). The *I–V* relationship is shown in Fig. 6 *B*. Control cells dialysed with Na^+^‐containing external and pipette solutions generated *I*_CRAC_ (Fig. 6 *A*) with a very similar *I–V* relationship (Fig. 6 *B*) and a similar peak amplitude (Fig. 6 *C*) to that seen when the current was evoked in Na^+^‐free solutions. Therefore, despite extensive dialysis with Na^+^‐free pipette solution in the absence of external Na^+^, *I*_CRAC_ development is not impaired in weak intracellular Ca^2+^ buffer.

### *I*_CRAC_ activation under physiological conditions of weak intracellular Ca^2+^ buffering is independent of intra‐ and extracellular Na^+^

**Figure 7:**
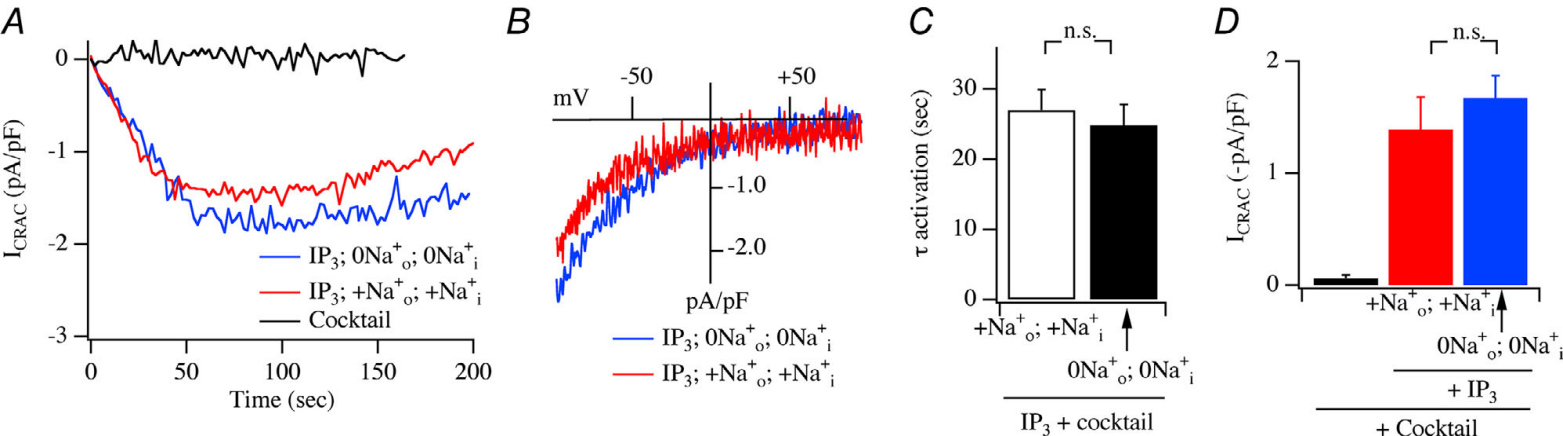
Impact of removing extracellular Na^+^ on *I*_CRAC_ activated by Ins*P*_3_ in weak intracellular Ca^2+^ buffer and energized mitochondria *A*, time course of *I*_CRAC_ compared between a cell exposed to Na^+^‐containing external and pipette solutions (red trace) and a cell dialysed with Na^+^‐free solution and placed in Na^+^‐free external solution (16 min before break‐in; blue trace). In both cases, pipette solution contained weak Ca^2+^ buffer, mitochondrial cocktail and Ins*P*_3_. Dialysis with a Na^+^‐containing pipette solution containing weak Ca^2+^ buffer and cocktail without Ins*P*_3_ failed to activate a current (black trace). *B*, *I–V* curves taken when the currents in panel *A* had reached steady state. *C*, the activation time constant compared for the different conditions. There were no statistically significant differences between the groups. *D*, bar chart plotting amplitude of *I*_CRAC_ for the conditions shown. Cocktail is mean of 7 cells, ${\mathrm{Na}}_{0}^{+}/{\mathrm{Na}}_{i}^{+}$ 9 cells and $0\;{\mathrm{Na}}_{0}^{+}/0\;{\mathrm{Na}}_{i}^{+}$ 10 cells. There was no significant difference between Ins*P*_3_ groups. Both these groups were significantly different from cocktail alone (*P* < 0.01 in each case). Cocktail solution without Ins*P*_3_ was ^+^Na^+^_o_; ^+^Na^+^_i_.

Under physiological conditions of weak intracellular Ca^2+^ buffering, Ins*P*_3_ activates *I*_CRAC_ provided mitochondrial Ca^2+^ uptake is maintained by inclusion of the mitochondrial cocktail (Gilabert & Parekh, 2000). To test whether intra‐ or extracellular Na^+^ was required to support *I*_CRAC_ under physiological conditions of weak buffering and with Ins*P*_3_ as the stimulus to deplete stores, we examined the ability of Ins*P*_3_ to activate the current in the absence of both extra‐ and intracellular Na^+^. Dialysis with Ins*P*_3_ activated *I*_CRAC_ in Na^+^‐containing external and pipette solutions (Fig. 7 *A*). The *I–V* relationship was typical of *I*_CRAC_ (Fig. 7 *B*). *I*_CRAC_ activated with a mono‐exponential time constant (Fig. 7 *C*) to reach a peak amplitude of ∼−1.5 pA/pF (Fig. 7 *D*). This is significantly less than that seen in strong buffer (Fig. 1 *D*) and reflects incomplete store depletion due to active SERCA pumps (Glitsch *et al*. 2002b). Robust *I*_CRAC_ also developed in cells exposed to Na^+^‐free extracellular solution and dialysed with a Na^+^‐free pipette solution (Fig. 7 *A*), and the *I–V* relationship (Fig. 7 *B*), the activation kinetics (Fig. 7 *C*) and the amplitude of the current (Fig. 7 *D*) were all similar to those seen in Na^+^‐containing solutions. Dialysis with a weak buffer‐based internal solution containing cocktail but no Ins*P*_3_ failed to activate any inward current (Fig. 7 *A* and *D*), confirming the development of the current was due to store depletion by Ins*P*_3_.

### Store‐operated Ca^2+^ entry in intact cells in the absence of external Na^+^

**Figure 8:**
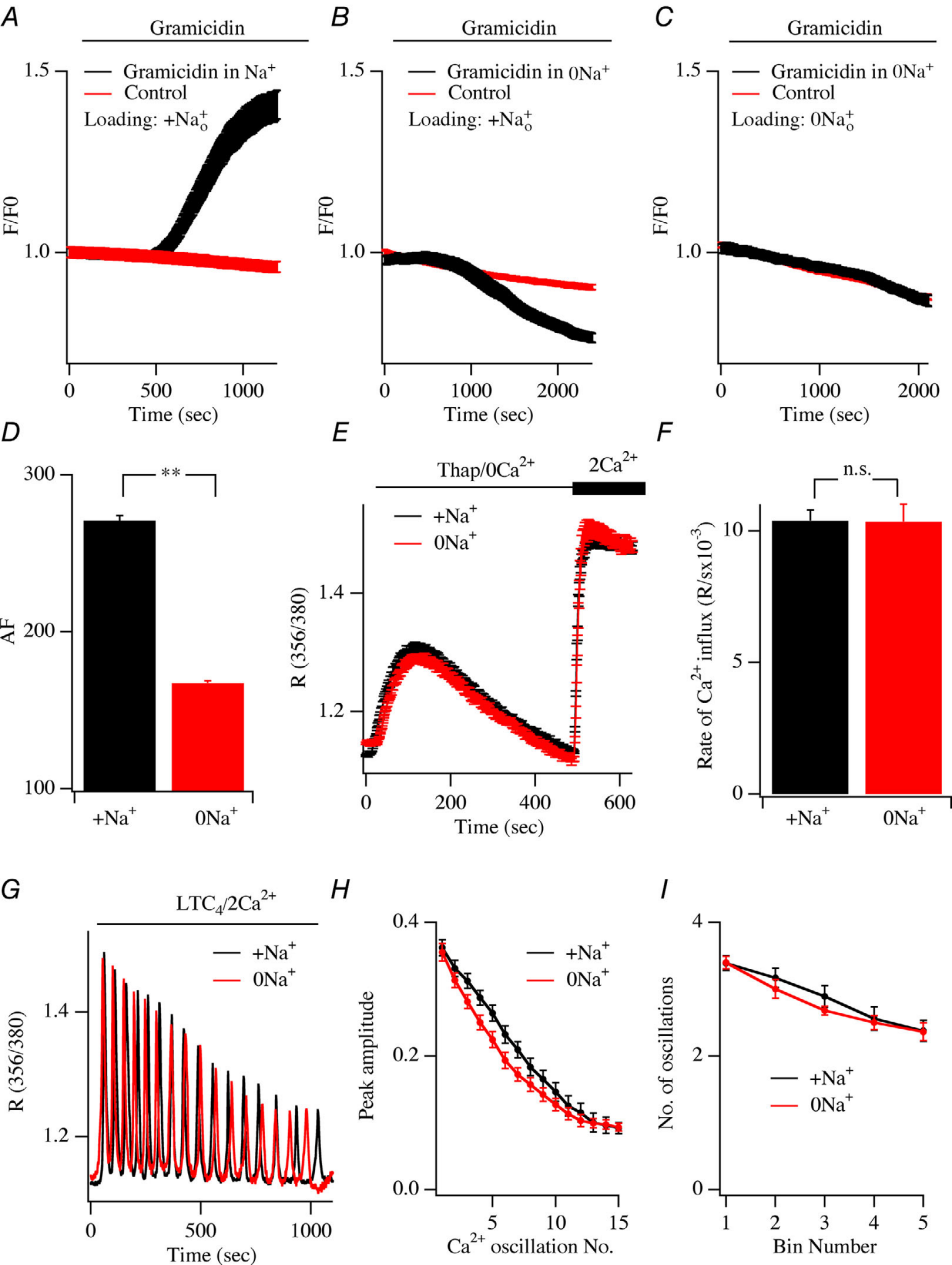
Effect of removing external Na^+^ on store‐operated Ca^2+^ entry and cytosolic Ca^2+^ oscillations *A*, application of the Na^+^ ionophore gramicidin A increases cytosolic Na^+^ when applied in Na^+^‐containing extracellular solution to CoroNa green‐loaded cells (black trace, mean of 9 cells). The red trace (mean of 7 cells) shows the linear decrease in CoroNa green fluorescence in non‐stimulated (control) cells. *B*, black trace (mean of 7 cells) shows the change in cytosolic Na^+^ in cells loaded with dye in Na^+^‐containing solution but exposed to Na^+^‐free external solution just prior (∼2 min) to gramicidin exposure. The red trace (mean of 10 cells) is the decay in fluorescence in non‐stimulated (control) cells. *C*, black trace (mean of 12 cells) shows the change in cytosolic Na^+^ in cells loaded with dye in Na^+^‐free external solution and then maintained in Na^+^‐free external solution during gramicidin exposure. The red trace (mean of 9 cells) is the decay in fluorescence in non‐stimulated cells and superimposes on the black one. *D*, bar chart comparing absolute CoroNa green fluorescence in control cells after loading in Na^+^‐containing external solution (black bar, mean of 9 cells) or after loading and then being kept in Na^+^‐free solution (red bar, mean of 9 cells). The *y*‐axis denotes absolute fluorescence (AF). *E*, store‐operated Ca^2+^ entry, in fura 2‐loaded cells, compared with cells loaded in and then maintained in either Na^+^‐containing (20 cells) or Na^+^‐free (25 cells) external solution. *F*, aggregate data comparing the rate of Ca^2+^ entry from experiments as in panel *E*. There was no statistically significant difference between the 2 groups (*P* > 0.1). *G*, cytosolic Ca^2+^ oscillations are shown in response to LTC_4_ stimulation for a cell loaded in and then maintained in Na^+^‐containing external solution and a cell loaded in and maintained in Na^+^‐free external solution, as indicated. *H*, graph comparing the amplitude of each Ca^2+^ oscillation in the presence or absence of external Na^+^. Each condition is the mean of between 12 and 15 cells. *I*, the number of cytosolic Ca^2+^ oscillations compared for the conditions shown. Oscillations were measured in bin numbers of 200 s.

In fura 2‐loaded cells, stimulation with thapsigargin in Ca^2+^‐free external solution leads to a transient rise in cytosolic Ca^2+^ as Ca^2+^ is released from the stores. Readmission of external Ca^2+^, once Ca^2+^ release has terminated, results in a large rise in cytosolic Ca^2+^ due to Ca^2+^ entry through CRAC channels (Bird & Putney, 1993). We compared the rate of rise and extent of the cytosolic Ca^2+^ rise following Ca^2+^ entry through store‐operated channels in intact cells in the presence or absence of extracellular Na^+^. To ensure intracellular Na^+^ was reduced following the removal of external Na^+^, we loaded cells with fura 2 in Na^+^‐free external solution and then maintained the cells in Na^+^‐free solution. Cells were therefore exposed continuously to Na^+^‐free solution for >1 h prior to recording. We checked whether this protocol indeed lowered cytosolic Na^+^. To this end, cells were loaded with the Na^+^‐sensitive fluorescent dye CoroNa green in either the presence or the absence of external Na^+^ and then subsequently challenged with the Na^+^ ionophore gramicidin A. Gramicidin A aggregates slowly in the plasma membrane to form Na^+^‐permeable ion channels. In cells loaded with CoroNa green in the continuous presence of extracellular Na^+^, application of gramicidin A led to a prominent rise in cytosolic Na^+^ due to Na^+^ entry (Fig. 8 *A*). No such increase was seen in control cells not exposed to gramicidin A (Fig. 8 *A*). In cells that were loaded with CoroNa green in the presence of extracellular Na^+^ but were then exposed to Na^+^‐free solution briefly (∼2 min) before challenge with gramicidin A, no rise in cytosolic Na^+^ occurred (Fig. 8 *B*). Cytosolic Na^+^ in fact declined somewhat in these cells after exposure to gramicidin A as cytosolic Na^+^ was initially higher than the Na^+^ concentration in Na^+^‐free extracellular solution and therefore exited the cell through the gramicidin A channels in the plasma membrane (Fig. 8 *B*). If cells were loaded with CoroNa green in Na^+^‐free extracellular solution and then maintained in Na^+^‐free solution, gramicidin A exposure failed to alter cytosolic Na^+^ (Fig. 8 *C*), consistent with these cells having significantly reduced cytosolic Na^+^. We compared the absolute levels of CoroNa green intensity in cells loaded in Na^+^‐containing external solution with those loaded in Na^+^‐free solution. The absolute fluorescence was significantly lower in the latter cells (Fig. 8 *D*). Therefore cytosolic Na^+^ was considerably reduced in cells exposed to Na^+^‐free extracellular solution for >1 h, confirming that the use of Na^+^‐free solution during the loading process was indeed effective.

In cells loaded with fura 2 in the continuous presence of extracellular Na^+^, stimulation with thapsigargin evoked Ca^2+^ release from the stores followed by prominent store‐operated Ca^2+^ entry (Fig. 8 *E*; aggregate data summarized in Fig. 8 *F*). In cells loaded in Na^+^‐free solution, a condition that lowers cytosolic Na^+^ (Fig. 8 *D*), both Ca^2+^ release and store‐operated Ca^2+^ entry were not significantly different from corresponding responses obtained in the presence of extracellular Na^+^ (Fig. 8 *E* and *F*).

In some cell types, a rise in cytosolic Ca^2+^ activates non‐selective TRPM4 leading to membrane depolarization (Vennekens *et al*. 2007; Holzmann *et al*. 2015). This reduces Ca^2+^ influx and constitutes a negative feedback mechanism for regulation of store‐operated Ca^2+^ entry (Launay *et al*. 2004). Removal of extracellular Na^+^ would therefore be expected to enhance store‐operated Ca^2+^ influx. However, we found that removal of Na^+^ had no effect on the rate or extent of Ca^2+^ influx (Fig. 8 *E* and *F*). Two pieces of evidence argue against a role for TRPM4 in regulating CRAC channels under our conditions. First, dialysis of RBL cells with pipette solutions containing free Ca^2+^ in the range 300 nM to 2 μM, well within the range for robust activation of TRPM4 channels (Launay *et al*. 2002), failed to activate any current in the presence or absence of external Na^+^ (Bakowski and Parekh, unpublished data). Second, only *I*_CRAC_ develops following dialysis with a pipette solution containing Ins*P*_3_ and thapsigargin to deplete stores and 2 mM ATP as the only exogenous Ca^2+^ buffer (Fierro and Parekh, 2000). Under these conditions, free Ca^2+^ is weakly buffered at ∼1 μM and will increase further upon CRAC channel activation. Nevertheless, only the Ca^2+^‐selective CRAC current developed in the presence of external Na^+^.

### Cytosolic Ca^2+^ oscillations are unaffected by removal of extracellular Na^+^

Physiological Ca^2+^ signals are often generated following stimulation of Gq‐coupled receptors and are presented to cells in the form of repetitive cytosolic Ca^2+^ oscillations (Thomas *et al*. 1996). Activation of cysteinyl leukotriene type 1 receptors in RBL cells with the agonist leukotriene C_4_ (LTC_4_) evokes repetitive cytosolic Ca^2+^ oscillations, which arise from regenerative Ca^2+^ release from Ins*P*_3_‐sensitive Ca^2+^ stores followed by Ca^2+^ entry through CRAC channels (Di Capite *et al*. 2009). The Ca^2+^ entry component refills the stores (Di Capite *et al*. 2009) and repletes phosphatidylinositol 4,5‐bisphosphate levels in readiness for the next oscillatory cycle (Alswied & Parekh, 2015). The Ca^2+^ oscillations run down quickly in the absence of external Ca^2+^, following knock down of Orai1 or after pharmacological block of CRAC channels (Kar *et al*. 2012a). If Na^+^ entry is required for sustaining CRAC channel activity, a prediction would therefore be that Ca^2+^ oscillations evoked by leukotriene receptor stimulation should also run down quickly in Na^+^‐free external solution as CRAC channel activity should be compromised. To test this, we compared the amplitude and frequency of cytosolic Ca^2+^ oscillations generated in response to LTC_4_ stimulation in cells maintained either in the presence or in the absence of external Na^+^. Numerous Ca^2+^ oscillations were observed in the presence of extracellular Na^+^ (Fig. 8 *G*), and the amplitude and number of oscillations gradually declined over time (Fig. 8 *H* and *I*), due to receptor desensitization (Ng *et al*. 2012). In cells loaded and then maintained in Na^+^‐free solution for >1 h, LTC_4_ still elicited numerous Ca^2+^ oscillations (Fig. 8 *G*) and the amplitude and number of oscillations were similar to those obtained in the presence of Na^+^ (Fig. 8 *H* and *I*).

### CRAC channel‐dependent gene expression is unaffected by removal of Na^+^

**Figure 9:**
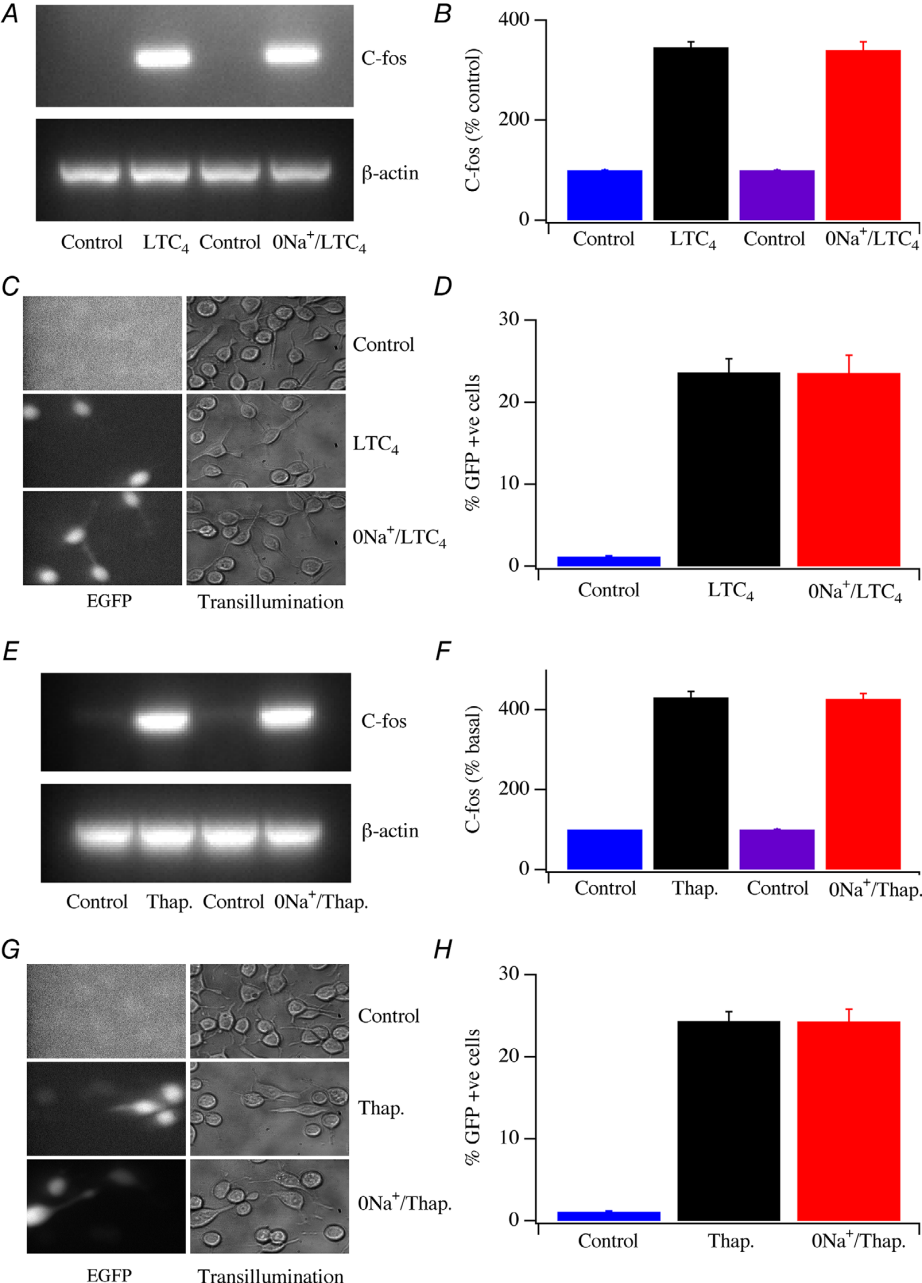
CRAC channel‐dependent gene expression is unaffected by removing external Na^+^ *A*, c‐fos expression compared between cells stimulated with LTC_4_ (160 nM) in Na^+^‐containing external solution and cells stimulated in Na^+^‐free solution. *B*, aggregate data compared from 3 independent experiments. For stimulation in Na^+^‐free solution, cells were exposed to Na^+^‐free solution for 1 h and then kept in this solution during and after stimulation. *C*, images comparing NFAT–GFP reporter gene expression at rest and then after 10 min stimulation with LTC_4_ in the presence or absence of external Na^+^. *D*, aggregate data from experiments as in panel *C* compared. Cells were kept in Na^+^‐free solution for 1 h prior to LTC_4_ challenge and then maintained in Na^+^‐free solution both during stimulation and then after stimulation for a further 30 min before cells were returned to DMEM (see Methods). *E*, gel comparing c‐fos expression following stimulation with thapsigargin (0.1 μM;8 min) under the conditions shown. *F*, aggregate data compared from 2 independent experiments as in panel *E*. *G*, NFAT–GFP reporter gene expression compared for the conditions shown. *H*, aggregate data compared from experiments as in panel *G*.

Ca^2+^ microdomains near open CRAC channels activate the transcription factors c‐fos and NFAT1 through recruitment of local signalling pathways (Ng *et al*. 2009; Kar *et al*. 2014). Although *I*_CRAC_, store‐operated Ca^2+^ entry and agonist‐evoked physiological Ca^2+^ signalling were all unaffected by removal of external Na^+^ and the subsequent reduction in cytosolic Na^+^, we considered the possibility that cytosolic Na^+^ regulated CRAC channel‐driven responses over longer time periods. To test this, we measured Ca^2+^‐dependent gene expression following CRAC channel activation. Stimulation with LTC_4_ for 10 min in the presence of external Na^+^ evoked a robust increase in the expression of c‐fos (Fig. 9 *A*). Exposure to Na^+^‐free solution for >1 h prior to challenge with LTC_4_ in Na^+^‐free solution also resulted in an increase in c‐fos that was not significantly different in size from the response obtained in Na^+^‐containing solution (Fig. 9 *A*; aggregate data are summarized in Fig. 9 *B*). We also measured NFAT‐driven gene expression using a GFP reporter gene under the control of an NFAT promoter (Kar *et al*. 2012b). Stimulation with LTC_4_ for 10 min in the presence of external Na^+^ resulted in GFP expression 24 h later (Fig. 9 *C* and *D*). A similar level of reporter gene expression was obtained when cells were exposed to Na^+^‐free solution for >1 h before stimulation with LTC_4_ in Na^+^‐free solution (Fig. 9 *C* and *D*). Identical results were obtained when we used a strong stimulus to maximally activate CRAC channels. Stimulation with thapsigargin activated c‐fos (Fig. 9 *E* and *F*) and NFAT reporter gene expression (Fig. 9 *G* and *H*) both in the presence and in the absence of external Na^+^, and to similar extents.

### MCU is required for *I*_CRAC_ development under physiological conditions

**Figure 10:**
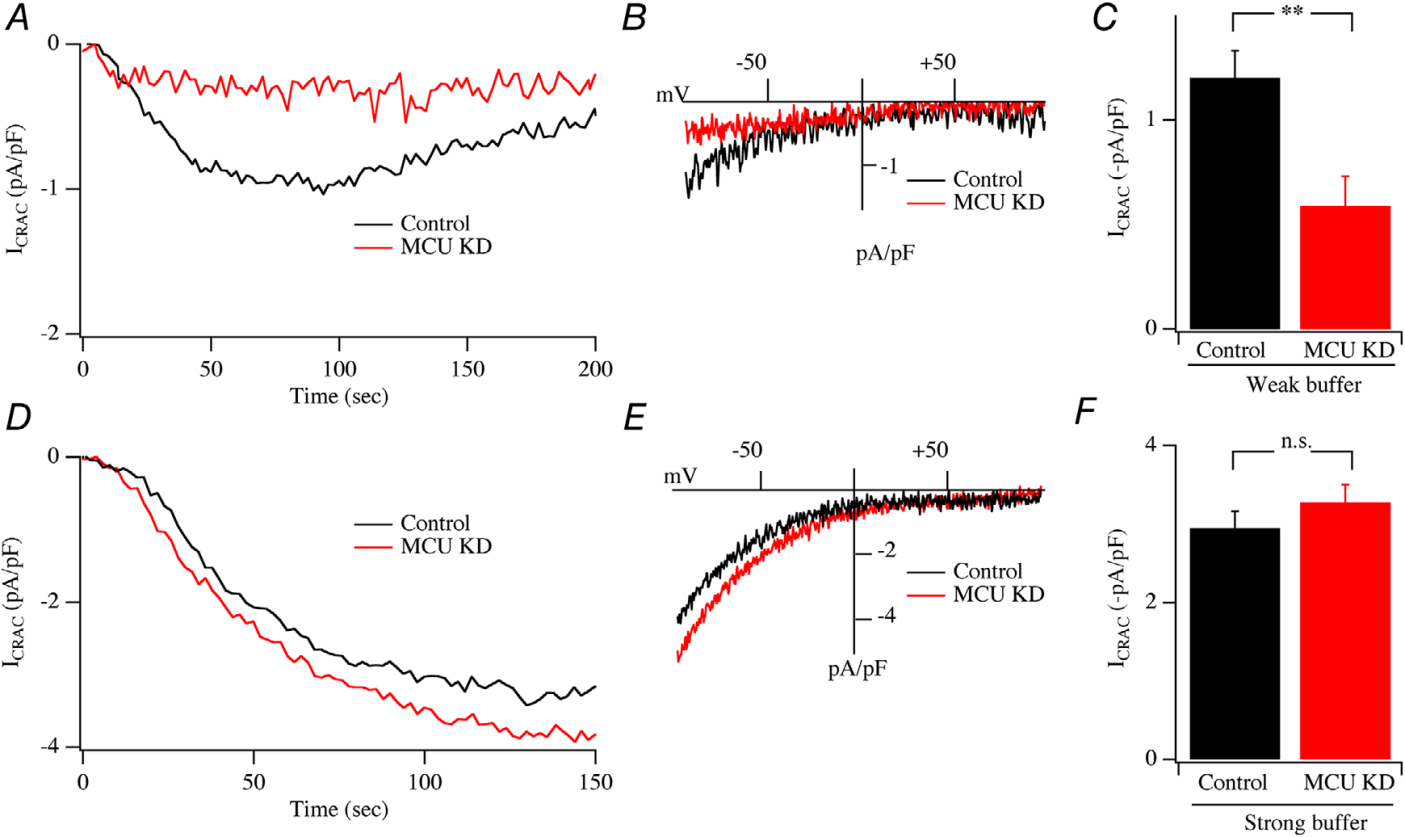
Effect of knockdown of the MCU channel on *I*_CRAC_ activation *A*, development of *I*_CRAC_ compared between a control cell and one in which the MCU had been knocked down. *B*, *I–V* curves from panel *A* (taken after ∼100 s). *C*, amplitude of *I*_CRAC_ compared for the conditions shown. In panels *A–C*, cells were dialysed with a Na^+^‐containing pipette solution supplemented with Ins*P*_3_, weak Ca^2+^ buffer and mitochondrial cocktail. External solution contained Na^+^. *D*, development of *I*_CRAC_ compared between a control cell and one in which the MCU had been knocked down, but now for cells dialysed with strong Ca^2+^ buffer (10 mM EGTA), together with Ins*P*_3_ and cocktail. *E*, *I–V* curves from panel *D*. *F*, amplitude of *I*_CRAC_ compared for the conditions indicated. In panels *D–F*, cells were dialysed with a Na^+^‐containing pipette solution supplemented with Ins*P*_3_, strong Ca^2+^ buffer (10 mM EGTA) and mitochondrial cocktail. Data shown are the average of 8 control cells and 9 MCU KD cells (weak Ca^2+^ buffer) and 7 control cells and 8 MCU KD cells (strong Ca^2+^ buffer).

Mitochondrial Ca^2+^ uptake is necessary for the development of *I*_CRAC_ under physiological conditions of weak intracellular Ca^2+^ buffering. To examine whether the MCU channel was involved, we compared the size of the current in control cells with that in cells in which the MCU had been knocked down using a siRNA‐based strategy. MCU knockdown reduced the rate of Ca^2+^ entry following readmission of external Ca^2+^ to thapsigargin‐treated cells by 71 ± 5%, consistent with our previous study using the same siRNA construct (Samanta *et al*. 2014). Dialysis with Ins*P*_3_ in weak Ca^2+^ buffer supplemented with mitochondrial cocktail evoked a clear *I*_CRAC_ in control cells (Fig. 10 *A*; *I–V* relationship is shown in Fig. 10 *B* and mean amplitude in Fig. 10 *C*). However, the current was significantly smaller following knockdown of the MCU (Fig. 10 *A* and *C*). If the role of the MCU under these conditions is to enable mitochondrial Ca^2+^ uptake, then one would expect this function to be obviated by dialysis with strong intracellular Ca^2+^ buffer as the latter would capture the vast majority of cytosolic Ca^2+^ and thereby render a role for mitochondrial Ca^2+^ uptake obsolete. Consistent with this, dialysis with a pipette solution containing Ins*P*_3_, mitochondrial cocktail and strong Ca^2+^ buffer (10 mM EGTA) activated robust *I*_CRAC_ (Fig. 10 *D* and *F*) that was unaffected by MCU knockdown with regards to time course of development, *I–V* relationship and peak amplitude (Fig. 10 *D*–*F*).

### Effect of NCLX knockdown on *I*_CRAC_

**Figure 11:**
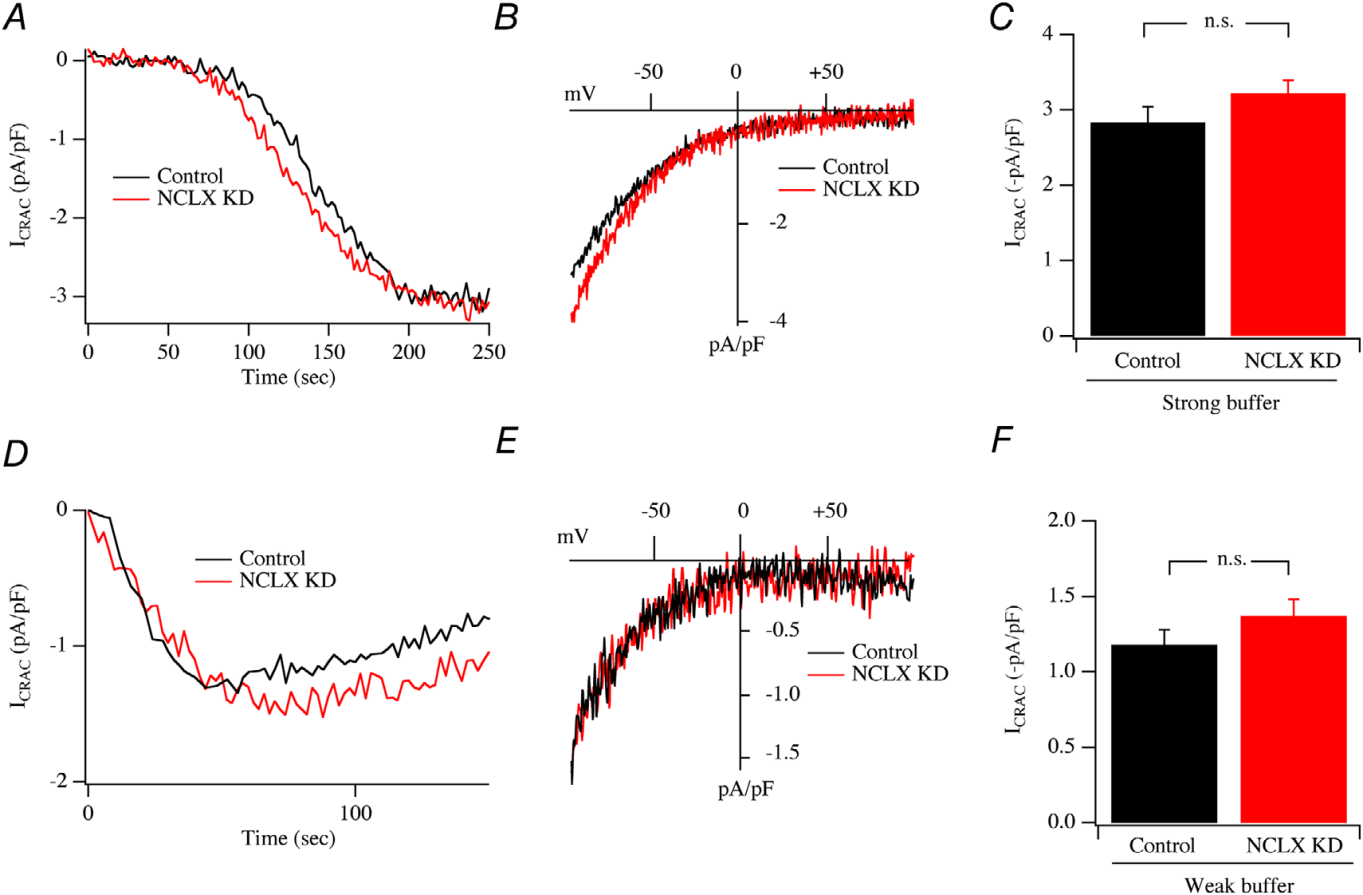
Effect of knockdown of NCLX protein on *I*_CRAC_ activation *A*, time course of *I*_CRAC_ compared between a control cell and one in which NCLX was knocked down. Cells were dialysed with a pipette solution containing 10 mM EGTA to deplete stores passively. *B*, *I–V* curves, taken from panel *A* at steady state, compared. *C*, bar chart comparing amplitude of *I*_CRAC_ for the conditions shown (each bar is mean of 6 cells). *D*, time course of *I*_CRAC_ shown for a control cell and for one in which NCLX had been knocked down. Pipette solution contained weak Ca^2+^ buffer, mitochondrial cocktail and Ins*P*_3_. *E*, *I–V* curves, taken from panel *D* at steady state. *F*, bar chart comparing amplitude of *I*_CRAC_ for the conditions shown (each bar is mean of 7 cells).

To assess the role of mitochondrial NCLX on *I*_CRAC_ activation, we used an siRNA‐based approach to knock down expression of the transporter. Western blot analysis showed NCLX knockdown was 61 ± 6%, similar to what we had observed previously in RBL cells using the same siRNA construct (Samanta *et al*. 2018). We activated *I*_CRAC_ in a similar way to that used by Ben‐Kassus Nissim *et al*. (2017), namely through passive depletion of stores by dialysis with strong buffer. Robust *I*_CRAC_ developed following dialysis with 10 mM EGTA in control recordings (Fig. 11 *A* and*B*), to reach an amplitude (Fig. 11 *C*) similar to that seen in Fig. 4. Knockdown of NCLX protein did not prevent the development of *I*_CRAC_, nor did it reduce the size of the current (Fig. 11 *A*–*C*). In fact, there was a tendency for the amplitude of the current to increase in NCLX‐deficient cells, although this was not statistically significant (Fig. 11 *C*). We repeated these experiments but now under more physiological conditions by using a pipette solution that contained weak Ca^2+^ buffer and mitochondrial cocktail together with Ins*P*_3_ to deplete the stores. *I*_CRAC_ activated in both control cells and in cells in which NCLX had been knocked down (Fig. 11 *D*–*F*). As was the case with strong buffer (Fig. 11 *C*), the current amplitude tended to be larger in NCLX knockdown cells (Fig. 11 *F*), although this was not significant (*P* = 0.1). Knockdown of NCLX did not compromise the development of *I*_CRAC_ in either strong or weak intracellular Ca^2+^ buffer.

## Discussion

Since the discoveries of STIM1 and Orai1, growing evidence attests to a central role for CRAC channels in controlling a plethora of spatially and temporally distinct signalling pathways (Parekh, 2010; Prakriya & Lewis, 2015). An understanding of the molecular mechanisms that regulate the channels will provide new insights into the function of store‐operated Ca^2+^ entry in a physiological context. Our results reveal that Ca^2+^ uptake by the MCU channel is required for the full development of *I*_CRAC_ under physiological conditions of intracellular Ca^2+^ buffering. We also find that the development of *I*_CRAC_ under a range of different conditions, of store‐operated Ca^2+^ entry, of cytosolic Ca^2+^ oscillations to a physiological trigger as well as CRAC channel‐dependent gene expression are all insensitive to changes in extracellular and intracellular Na^+^.

### Central role for the MCU channel

Although dialysis with Ins*P*_3_ activates *I*_CRAC_ in the presence of strong intracellular Ca^2+^ buffer (10 mM EGTA or BAPTA) in whole‐cell patch clamp recordings from RBL mast cells, the current does not develop when weak, physiological levels of buffer (0.1–0.35 mM) are used instead (Glitsch & Parekh, 2000). However, the current develops under these conditions when precautions are taken to ensure mitochondria are retained in an energized state by inclusion of a mitochondrial cocktail that maintains flux through the Krebs cycle (Gilabert & Parekh, 2000). The hyperpolarized potential across the inner mitochondrial membrane provides a large driving force for cytosolic Ca^2+^ entry into the matrix through the MCU channel, enabling more extensive store depletion in response to Ins*P*_3_ challenge as well as reducing cytosolic Ca^2+^‐dependent slow inactivation of the CRAC channels in RBL cells (Gilabert & Parekh, 2000; Glitsch *et al*. 2002a). This combination results in robust *I*_CRAC_ development. The MCU complex is composed of the pore‐forming MCU protein in combination with the essential subunit EMRE, MCUb and regulators MICU1 and MICU2 (Sancak *et al*. 2013; Mammucari *et al*. 2018). Our data demonstrate that knock‐down of the MCU protein inhibits the development of *I*_CRAC_ under physiological conditions of weak intracellular Ca^2+^ buffering. However, *I*_CRAC_ develops fully in cells in which MCU has been knocked down when strong Ca^2+^ buffer is used, revealing that the role of MCU indeed involves mitochondrial Ca^2+^ uptake and therefore buffering of cytosolic Ca^2+^. This central role for the pore‐forming MCU subunit in supporting *I*_CRAC_ under physiological conditions helps explain why cytosolic Ca^2+^ oscillations evoked by receptor stimulation run down quickly in MCU‐deficient cells (Samanta *et al*. 2014). The loss of mitochondrial Ca^2+^ buffering accelerates Ca^2+^‐dependent slow inactivation of CRAC channels and thereby compromises store refilling, needed to maintain the oscillatory Ca^2+^ response. Mitochondrial Ca^2+^ uptake is essential for supporting CRAC channel activity under physiological conditions in other cell types including T cells (Hoth *et al*. 1997; Hoth *et al*. 2000; Quintana *et al*. 2007) and endothelia (Malli *et al*. 2003) where it is likely, in light of our data, to also require the MCU channel.

### CRAC channels do not require extracellular or internal Na^+^ for activation

The unitary conductance of the CRAC channel is tiny, in the range of a few femtosiemens (Zweifach & Lewis, 1993), and is well beyond the bandwidth of current patch clamp amplifiers. Selectivity and gating properties of the channels perforce have been derived from analysis of the whole‐cell current. Pioneering work on the biophysical properties of *I*_CRAC_ in mast cells, RBL cells, T lymphocytes and Jurkat T cells established several common features including high selectivity for Ca^2+^ over other cations including Na^+^, steep inward rectification, a very positive reversal potential (>+80 mV) and a very low unitary chord conductance (reviewed in Parekh & Penner, 1997). In fact, the selectivity of CRAC channels for Ca^2+^ over Na^+^ in various cell types has been found to be >1000:1 (Hoth, 1995), at least as high as that exhibited by voltage‐gated Ca^2+^ channels. These biophysical studies have provided an essential rulebook in assessing whether candidate genes encode the CRAC channel and played a central role in establishing that Orai1 comprised the CRAC channel pore (Prakriya & Lewis, 2015).

Recently, the unique Ca^2+^ selectivity of the CRAC channel has been questioned by the finding that store‐operated Na^+^ entry is required for the development of *I*_CRAC_ (Ben‐Kassus Nissim *et al*. 2017). In that study, removal of external Na^+^ led to a substantial reduction in the size of *I*_CRAC_ in RBL cells by ∼70% and abolished the endogenous current completely in HEK293 cells. It was suggested that store‐operated Na^+^ entry was essential for the full development of *I*_CRAC_ because the Na^+^ entry raised cytosolic Na^+^, which was then taken into the mitochondria in exchange for matrix Ca^2+^ through the mitochondrial NCLX transporter. Removal of matrix Ca^2+^ apparently prevented production of mitochondrial reactive oxygen species (ROS) and mitochondrially derived ROS was reported to inhibit CRAC channels by oxidizing Cys195 on the extracelluar side of the channels in the plasma membrane. The molecular basis of the store‐operated Na^+^ flux was unclear but was suggested to reflect permeation through store‐operated Na^+^‐permeable TRP channels, which would have to be activated by store depletion simultaneously with CRAC channels (Ben‐Kassus Nissim *et al*. 2017). Alternatively, the Na^+^ flux could reflect permeation through Orai1 protein. Regardless of the precise mechanism, the reported extracellular store‐operated Na^+^ flux contemporaneous with, and essential for, *I*_CRAC_ activation would mean that whole‐cell *I*_CRAC_ recordings have a Na^+^ component, challenging earlier interpretations of selectivity.

If the whole‐cell store‐operated current arose from two parallel currents, composed of *I*_CRAC_ and a Na^+^‐permeable current, then in addition to amplitude and time course of the current, key biophysical features such as the extent of inward rectification, the reversal potential of the current and the rate and extent of Ca^2+^‐dependent fast inactivation should be different in the absence of external Na^+^. Our new data, based on systematic removal of external Na^+^, internal Na^+^ or both, demonstrate that neither extracellular nor intracellular Na^+^ has any effect on the size of *I*_CRAC_, the extent of inward rectification, the time constant of activation, the reversal potential of the current or Ca^2+^‐dependent inactivation in RBL cells. In a human salivary gland cell line, a detailed study has revealed that Ca^2+^ entry through CRAC channels leads to insertion of TRPC1 channels into the plasma membrane (Cheng *et al*. 2011). *I*_CRAC_ activates first and this is followed by dramatic changes in the whole‐cell current as inserted TRPC1 channels then contribute. These changes include (i) an increase in the size of the whole‐cell current, (ii) a shift in the *I–V* relationship of the whole‐cell current to show much less inward rectification, and (iii) a large leftward shift of ∼80 mV in the reversal potential of the current. These changes were not seen in our experiments following alterations in extracellular Na^+^, consistent with the absence of a Na^+^‐permeable current.

Removal of extracellular Na^+^ failed to affect any of the properties of *I*_CRAC_ that we have measured using Ins*P*_3_ or passive store depletion (high EGTA or thapsigargin) to activate the current either in strong or weak Ca^2+^ buffer. The simplest explanation of our data is that *I*_CRAC_ is a Ca^2+^‐selective current and its activation and maintenance in RBL cells does not require a parallel Na^+^ current across the plasma membrane. We considered the possibility that a Na^+^ current was essential for CRAC channel activation as reported but was so small that it failed to impact on any of the hallmarks of *I*_CRAC_ that we have measured. Calculations suggest this is very unlikely. The NCLX has a *K*_M_ for cytosolic Na^+^ of ∼10 mM (Palty *et al*. 2010). In our experiments on RBL cells and in those reported in HEK cells (Ben‐Kassus Nissim *et al*. 2017), *I*_CRAC_ was activated by passive store depletion using a Na^+^‐free pipette solution and our ^23^Na NMR analysis confirmed we indeed used Na^+^‐free solution. As the cytosol was extensively dialysed before *I*_CRAC_ developed, cytosolic Na^+^ would have been very low in our experiments. For a store‐operated Na^+^ current to develop in parallel with *I*_CRAC_ and raise cytosolic Na^+^ rapidly within a few seconds to at least 10 mM, which would be required to enable effective mitochondrial NCLX activity, a whole‐cell Na^+^ current of ∼−100 pA would be required (ignoring Na^+^ clearance by pumps). This is larger than the typical ∼−50 pA *I*_CRAC_ we have recorded in RBL cells and considerably larger than the ∼−1 pA store‐operated current reported from HEK cells (Ben‐Kassus Nissim *et al*. 2017).

Removal of external Na^+^ also had no effect on the cytosolic Ca^2+^ rise due to store‐operated Ca^2+^ entry, on cytosolic Ca^2+^ oscillations that require Ca^2+^ entry through CRAC channels or on CRAC channel‐dependent gene expression. In our patch clamp experiments, recordings were made after 15–40 min of continuous exposure to Na^+^‐free solution and the Ca^2+^ measurements were made after cells had been exposed to Na^+^‐free solution for up to 90 min. Our experiments with gramicidin A and CoroNa green confirmed that such protocols had lowered cytosolic Na^+^ considerably.

Interestingly, whole‐cell *I*_CRAC_ has also been measured over several minutes in Jurkat T cells using a pipette solution totally devoid of Na^+^ (Zweifach & Lewis, 1995*a*,*b*). These cells are substantially smaller than RBL cells and whole‐cell dialysis of Na^+^ will therefore be quick. The CRAC current developed normally in the T cells and showed little sign of inactivation, being maintained for tens of seconds in high intracellular Ca^2+^ buffer despite the absence of Na^+^ from the pipette solution. Moreover, noise analysis studies on CRAC channels were conducted in isotonic Ca^2+^‐containing extracellular solution lacking Na^+^ and with Na^+^‐free internal solution (Zweifach & Lewis, 1993). These recordings of *I*_CRAC_ from jurkat T lymphocytes in the absence of extracellular and intracellular Na^+^ are consistent with the detailed biophysical analysis presented here for RBL cells.

### Implications for the involvement of mitochondrial Ca^2+^ transporters in regulating *I*_CRAC_ in RBL cells

Our patch clamp data obtained under physiological conditions of weak intracellular Ca^2+^ buffering, active SERCA pumps and energized mitochondria demonstrate that Ca^2+^ uptake by the MCU channel is required for full development of *I*_CRAC_. These data are in good agreement with previous studies that established mitochondrial Ca^2+^ buffering sustained store‐operated Ca^2+^ entry (Gilabert & Parekh, 2000; Hoth *et al*. 2000; Glitsch *et al*. 2002b) and we now show that the MCU is the route for mitochondrial Ca^2+^ uptake. The electrogenic mitochondrial NCLX protein is another important component of the Ca^2+^ handling machinery in the inner mitochondrial membrane which, when operating in forward mode, extrudes Ca^2+^ from the matrix in exchange for cytosolic Na^+^ (Palty *et al*. 2010). In depolarized mitochondria, NCLX can reverse and extrude matrix Na^+^ in exchange for cytosolic Ca^2+^ (Samanta *et al*. 2018). Because we took precautions to energize mitochondria, reverse mode NCLX activity does not contribute to mitochondrial Ca^2+^ uptake in our experiments.

NCLX operating in forward mode has been proposed to play an indispensable role in the development of *I*_CRAC_, taking up cytosolic Na^+^ that has been elevated by store‐operated Na^+^ entry in exchange for matrix Ca^2+^ (Ben‐Kassus Nissim *et al*. 2017). Our findings that development of *I*_CRAC_ in RBL cells in the presence of weak intracellular Ca^2+^ buffering depends on Ca^2+^ uptake by the MCU, that the current develops normally in the absence of both extracellular and intracellular Na^+^ and that *I*_CRAC_ develops normally after knockdown of NCLX demonstrate that Na^+^‐dependent fluxes across the plasma membrane and mitochondria are not essential for supporting *I*_CRAC_ at least in RBL cells. Mitochondrial Ca^2+^ buffering capacity is high with an estimated Ca^2+^ binding ratio of ∼4000 (Babcock *et al*. 1997) and therefore ∼40 times larger than the Ca^2+^ binding ratio of cytosol in most non‐excitable cells (Neher, 1995) including RBL cells, which have a Ca^2+^ binding ratio of ∼150 (Bakowski and Parekh, unpublished). Moreover, the major Ca^2+^ buffering system within the matrix is the formation of calcium phosphate complexes, with inorganic phosphate (P_i_) being rapidly taken up into the matrix through H_2_PO_4_^−^–H^+^ symport. The P_i_ effect on mitochondrial buffering has an EC_50_ of 0.096 mM (Wei *et al*. 2015). As we included 1 mM P_i_ in our mitochondrial cocktail, Ca^2+^ buffering within the matrix would have been maintained effectively in our experiments.

We have previously demonstrated using electron microscopy that mitochondria in RBL cells are located away from the plasma membrane, with very few within ∼100 nm of the cell periphery and most congregated ∼500–1000 nm away (Singaravelu *et al*. 2011). Therefore mitochondria buffer bulk cytosolic Ca^2+^ in RBL cells rather than local Ca^2+^ entry near the CRAC channels. Consistent with this, neither the rate nor extent of Ca^2+^‐dependent fast inactivation of CRAC channels, a process driven by Ca^2+^ microdomains that extend <10 nm from the channel pore (Zweifach & Lewis, 1995*a*), was affected by suppression of mitochondrial Ca^2+^ uptake (Gilabert & Parekh, 2000). The spatial segregation of mitochondria from CRAC channels at the plasma membrane explains why high concentrations of the slow Ca^2+^ chelator EGTA circumvent the need for the MCU channel in the activation of the current. It is important to stress that our findings are focused on RBL cells and we cannot rule out that mitochondrial NCLX plays an important role in supporting the small store‐operated current in HEK293 cells as reported (Ben‐Kassus Nissim *et al*. 2017), if mitochondria are close to the Ca^2+^ channels in this cell type so they can compete effectively with exogenous Ca^2+^ buffer. Ongoing work in our laboratory is addressing this.

## Additional information

### Competing interests

None declared.

### Author contributions

All electrophysiological studies, fluorescence measurements and siRNA knockdown were carried out in the Department of Physiology, Anatomy and Genetics. NMR experiments were conducted in the Department of Chemistry. All authors carried out the experiments for which the data are shown, and analysed and interpreted the data. All authors contributed to the writing of this study. All authors have read and approved the final version of this manuscript and agree to be accountable for all aspects of the work in ensuring that questions related to the accuracy or integrity of any part of the work are appropriately investigated and resolved. All persons designated as authors qualify for authorship, and all those who qualify for authorship are listed.

### Funding

This work was supported by an MRC UK Programme Grant to A.B.P. (LO1047X).
